# Head anatomy of a lantern shark wet‐collection specimen (Chondrichthyes: Etmopteridae)

**DOI:** 10.1111/joa.13822

**Published:** 2023-01-25

**Authors:** Manuel Andreas Staggl, Bernhard Ruthensteiner, Nicolas Straube

**Affiliations:** ^1^ Department of Biology II Ludwig‐Maximilians‐Universität München München Germany; ^2^ SNSB‐Bavarian State Collection of Zoology Munich Germany; ^3^ Department of Palaeontology, Faculty of Earth Sciences, Geography and Astronomy University of Vienna Vienna Austria; ^4^ Vienna Doctoral School of Ecology and Evolution (VDSEE) University of Vienna Vienna Austria; ^5^ Department of Natural History University Museum of Bergen Bergen Norway

**Keywords:** ampullae of Lorenzini, Chondrichthyes, deep sea, *Etmopterus lucifer*, micro‐CT

## Abstract

In this study, we apply a two‐step (untreated and soft tissue stained) diffusible iodine‐based contrast‐enhanced micro‐computed tomography array to a wet‐collection Lantern Shark specimen of *Etmopterus lucifer*. The focus of our scanning approach is the head anatomy. The unstained CT data allow the imaging of mineralized (skeletal) tissue, while results for soft tissue were achieved after staining for 120 h in a 1% ethanolic iodine solution. Three‐dimensional visualization after the segmentation of hard as well as soft tissue reveals new details of tissue organization and allows us to draw conclusions on the significance of organs in their function. Outstanding are the ampullae of Lorenzini for electroreception, which appear as the dominant sense along with the olfactory system. Corresponding brain areas of these sensory organs are significantly enlarged as well and likely reflect adaptations to the lantern sharks' deep‐sea habitat. While electroreception supports the capture of living prey, the enlarged olfactory system can guide the scavenging of these opportunistic feeders. Compared to other approaches based on the manual dissection of similar species, CT scanning is superior in some but not all aspects. For example, fenestrae of the cranial nerves within the chondrocranium cannot be identified reflecting the limitations of the method, however, CT scanning is less invasive, and the staining is mostly reversible and can be rinsed out.

## Graphical abstract

In this study, we present a two‐step micro‐computed tomography array to a 39‐year‐old wet‐collection Lantern Shark specimen of *Etmopterus lucifer*. The focus of our scanning approach is the head anatomy showing numerous internal structures, for example the ampullae of Lorenzini, the olfactory system and major brain areas. Significantly enlarged sensory systems likely reflect adaptations to the lantern sharks' deep‐sea habitat.

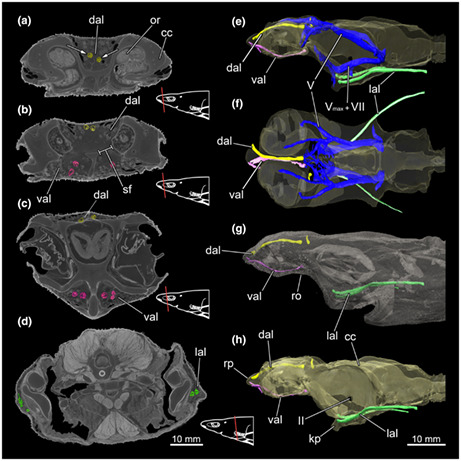

## 1 INTRODUCTION

More than half of the 543 shark species known today (Pöllerspoeck & Straube, 2021) are deep‐sea inhabitants (Kyne & Simpfendorfer, 2007). Most of the deep‐sea inhabiting species are squaliform sharks (Cotton & Grubbs, 2015). Currently, the order includes six families (Centrophoridae, Dalatiidae, Etmopteridae, Oxynotidae, Somniosidae and Squalidae) which occur in the deep seas of the tropical to boreal zones in the northern and southern hemispheres (Naylor et al., 2012). Due to sampling difficulties and scarcity of many species, the number of anatomical studies reaching beyond the analyses of skeletal elements is limited for most squaliforms (Ankhelyi et al., 2018; Denton et al., 2018; Maisey, 2004; Marinelli & Strenger, 1959; Mollen et al., 2012; Shirai, 1992a, 1992b; Shirai & Nakaya, 1990a, 1990b). Mostly, the easily accessible dogfish *Squalus acanthias* Linnaeus, 1758 has been widely used in anatomical soft tissue dissection studies in the past and became a model organism in vertebrate anatomy (De luliis & Pulera, 2019; Marinelli & Strenger, 1959). Shirai (1992b) performed comprehensive anatomical work for phylogenetic purposes over the entire phylogeny of Squaliformes. During this study, Shirai (1992b) examined a total of 87 species by dissection and established 173 derived characters used to reconstruct phylogenetic interrelationships. Within etmopterids, the velvet belly lantern shark, *Etmopterus spinax*, was in focus regarding the ultrastructural organization of photophores (Claes et al., 2010; Claes & Mallefet, 2008; Duchatelet et al., 2019).

Generally, non‐invasive methods are prefered for museum collection specimens over dissection. One option for non destructive anatomical examination of soft tissue of museum specimens is computed tomography (CT). Since hard tissue, for example calcified cartilage elements or teeth, are easily distinguishable using CT scanning, soft tissue poses a larger challenge, especially the differentiation of adjacent less calcified cartilage and soft tissue, as both tissue types have similar absorption spectra (Jeffery et al., 2011). Here, we overcome these issues by using diffusible iodine‐based contrast‐enhanced computed tomography (diceCT). This approach allows for distinguishing different tissue structures and types including challenging transitional areas using the absorption rate of iodine (Metscher, 2009). In this study, we present a non‐destructive approach for analysing the cranial anatomy of hard‐ and soft tissues in a lantern shark species for the first time and compare results to dissection‐based anatomical studies of Etmopteridae. New data on the anatomy of the cranial region of *Etmopterus lucifer* (Jordan & Snyder, 1902) are provided showing the structure and organization of skeletal and muscular elements as well as organs with cerebral innervation such as the ampullae of Lorenzini.

## 2 MATERIALS AND METHODS

### 2.1 Materials

The *Etmopterus lucifer* (ZSM‐30813) specimen used in this study is housed at the Bavarian State Collection of Zoology and was collected in 1979 SE of New Zealand (depth 700–720 m) by F. Pfeil. The specimen was fixed in 4% formaldehyde and is preserved in 70% EtOH solution.

### 2.2 Methods

#### 2.2.1 Staining

Only the head of the specimen was used in this study, therefore the specimen was decapitated with a scalpel. The head was immersed in a 1% (w/v) ethanolic iodine solution following the protocol by Metscher (2009). For the staining solution, 1 g I_2_ was dissolved in 100 ml denatured (MEK) 100% ethanol.

#### 2.2.2 CT acquisition and imaging procedures

Three CT acquisitions of the head were performed. One scan was done before immersing in the staining agent as a reference to retain the contrast of the calcareous skeletal elements. A second scan was performed after treatment with the iodine solution for 72 h. The final scan was performed after a total staining duration of 120 h. The sample—in ethanol or in staining solution‐soaked condition but surrounded by air—was mounted in a plastic container tightly closed with a lid to prevent drying out and stabilized by pieces of Styrofoam to prevent movements during the scanning process. CT acquisition was performed with a Phoenix Nanotom m (GE Sensing & Inspection). For scanning parameters, see Table 1. The final scan was carried out as ‘multiscan’ with three overlapping *z*‐direction positions.

**TABLE 1:** Scanning parameters applied for the CT acquisition

Name of project	Hours after immersion in staining solution	kV	mA	Total duration (min)	Voxel size (mm)	Filter	Number of projections
Etmop‐lucif‐ZSM‐30813	–	110	110	62	0.029	Cu 0.1 mm	1800
Etmop‐lucif‐ZSM‐30813_ai1	72	110	110	62	0.028	Cu 0.1 mm	1800
Etmop‐lucif‐ZSM‐30813_ai2mu	120	110	130	176	0.016	Cu 0.1 mm	3 × 1400

The CT reconstruction was conducted using phoenix datos|x2 software. The resulting 16‐bit volumes were converted to 8‐bit with VGStudio Max® (Volume Graphics) software at adjustment of the histogram. Further 3D graphic procedures (merging of the multiscan, orientation, segmentation, visualization, etc.) were performed with Amira® software (v. 6.4.0, Mercury Computer Systems, Inc.). The three portions of the multiscan were merged after manual registration and subsequent automatic refinement of the alignment. The resulting data set (2394 × 1856 × 4670 voxels) was resampled to half the original resolution. The unstained (Etmop‐lucif‐ZSM‐30813) and the 120 h stained (Etmop‐lucif‐ZSM‐30813_ai2mu) data sets were elastically co‐registered to clearly depict the position of the calcified elements in relation to the stained soft tissues (warped together). The 120 h stained data set was used for segmentation and subsequent surface rendering of organ components. Most segmentation steps were carried out ‘manually’ by subjective interpretation of structures on 1800 (cross) slices.

## 3 RESULTS

### 3.1 Comparison of CT data sets

**FIGURE 1:**
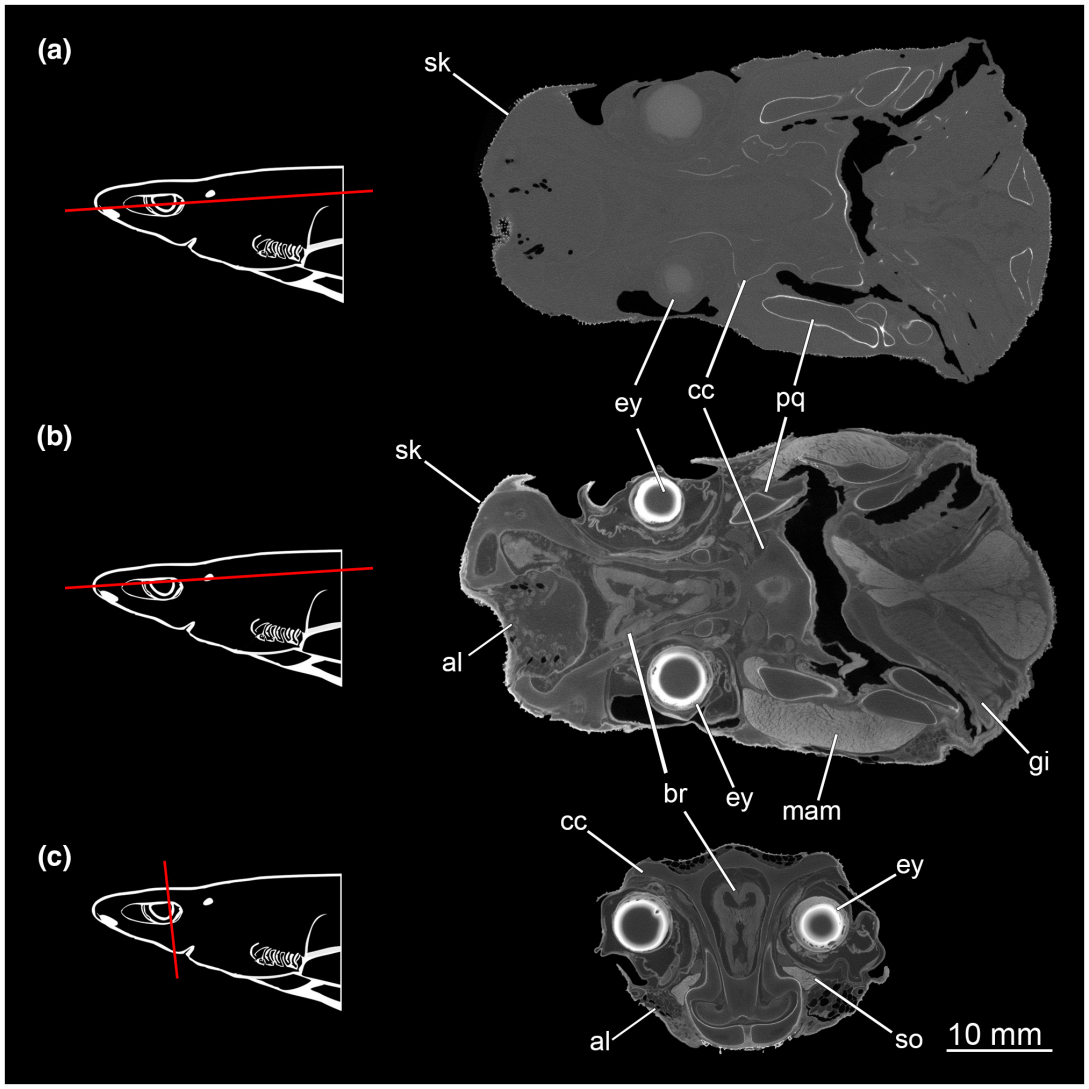
Comparison of CT scans of *Etmopterus lucifer* with and without contrasting in ethanolic iodine solution; the red line in the scheme indicates the plane from which the single image originates in the CT scan. (a) CT scan without staining (scan: Etmop‐lucif‐ZSM‐30813), coronal cut; (b) CT scan after 7 h staining time (scan: Etmop‐lucif‐ZSM‐30813_ai1), coronal cut; (c) CT scan after 120 h staining time (scan: Etmop‐lucif‐ZSM‐30813_ai2mu), transversal cut. Abbreviations: al, ampullae of Lorenzini; cc, chondrocranium; br, brain; ey, eye; g, gill; mam, *Musculus adductor mandibulae*; so, *Musculus suborbitalis*; pq, paltatoquadratum; sk, skin

The skin of the unstained head (Etmop‐lucif‐ZSM‐30813, Table 1) appears uniformly grey along with the underlying soft tissues against the much darker background. Only the calcified dermal denticles and some calcified parts of the cartilage skeletal elements are distinct, appearing in white colour. The dermal denticles are hook‐like and bent distally. After 72 h of immersion in the iodine solution (Etmop‐lucif‐ZSM‐30813_ai1, Table 1), the data reveal a significant contrast enhancement allowing better distinction of tissue types. After 120 h of staining, the skin also appears light grey but compared to the previous scan, no significant contrast enhancement on the inner soft tissues was observable (Etmop‐lucif‐ZSM‐30813_ai2mu, Table 1 and Figure 1).

The 120‐h data set allows a detailed description of the head anatomy. Contrast and resolution of the data set allowed for distinguishing many structures and tissue types, and, therefore, segmentation with color‐coding of different components.

#### 3.1.1 External morphology and skeletal elements head morphology

**FIGURE 2:**
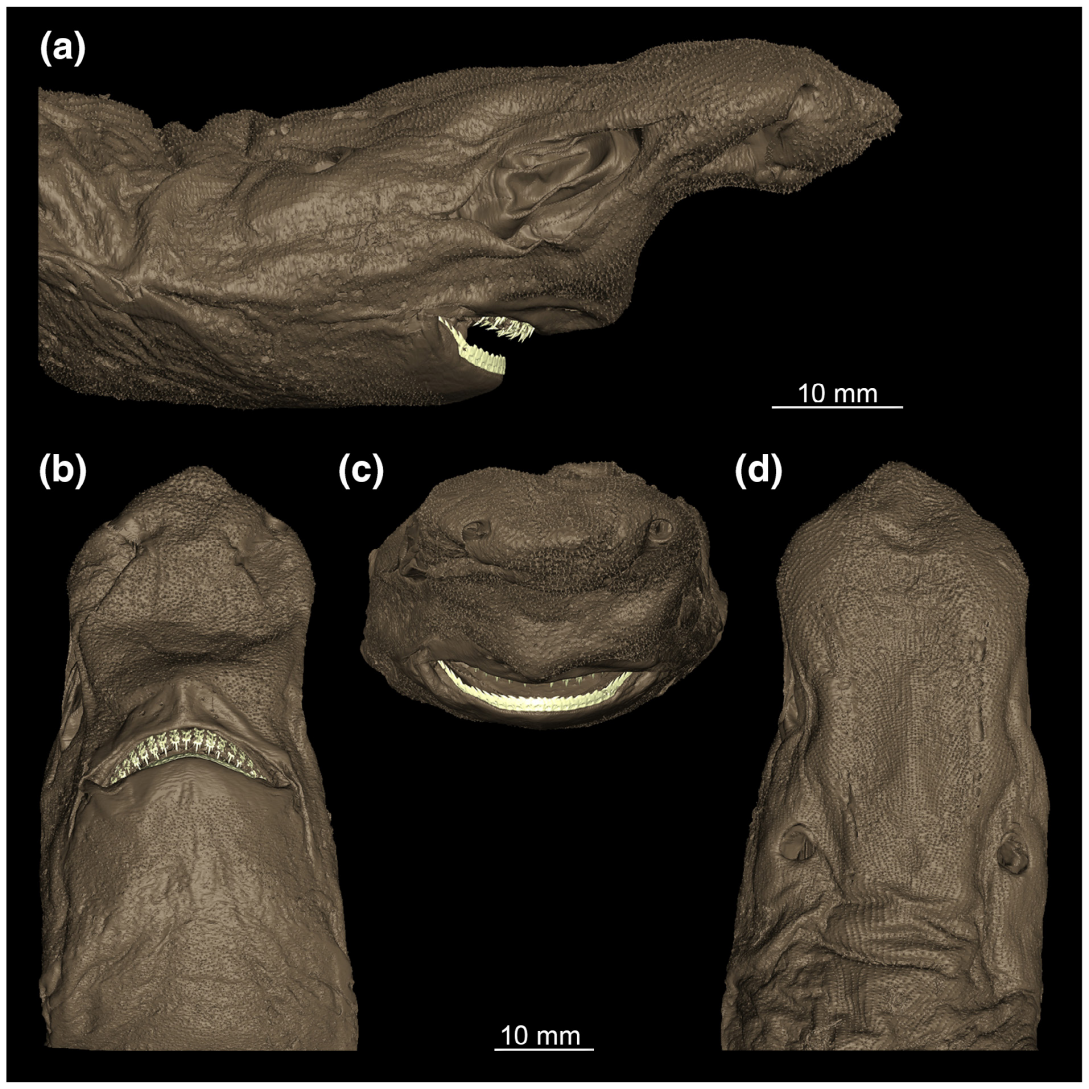
Different views of the outer surface of *Etmopterus lucifer* (a) lateral, (b) ventral, (c) posterior, (d) dorsal

The head of the *E. lucifer* specimen is elongated with a prominent rostrum (Figure 2). From a lateral view, the rostrum describes an arc of about 90° and tapers off at a flat angle to the rostral process (Figure 2a). The width of the rostrum corresponds approximately to the width of the orbital region and shows a tapering towards the rostral process anterior to the nasal capsules (Figure 2b,d). Rows of differently sized pores (pores of the ampullae of Lorenzini) on the lateral side of the head are running on the lateral side of the rostrum extending below the eyes dorsally and ventrally. The dentition can be seen in both the lower and upper jaws in the 3D model. The specimen displays typical characteristics of a collection specimen. It shows the typical shrinkage and wrinkling, and there is a loss of colouration, resulting in the specimen being brown instead of black.

#### 3.1.2 Chondrocranium

**FIGURE 3:**
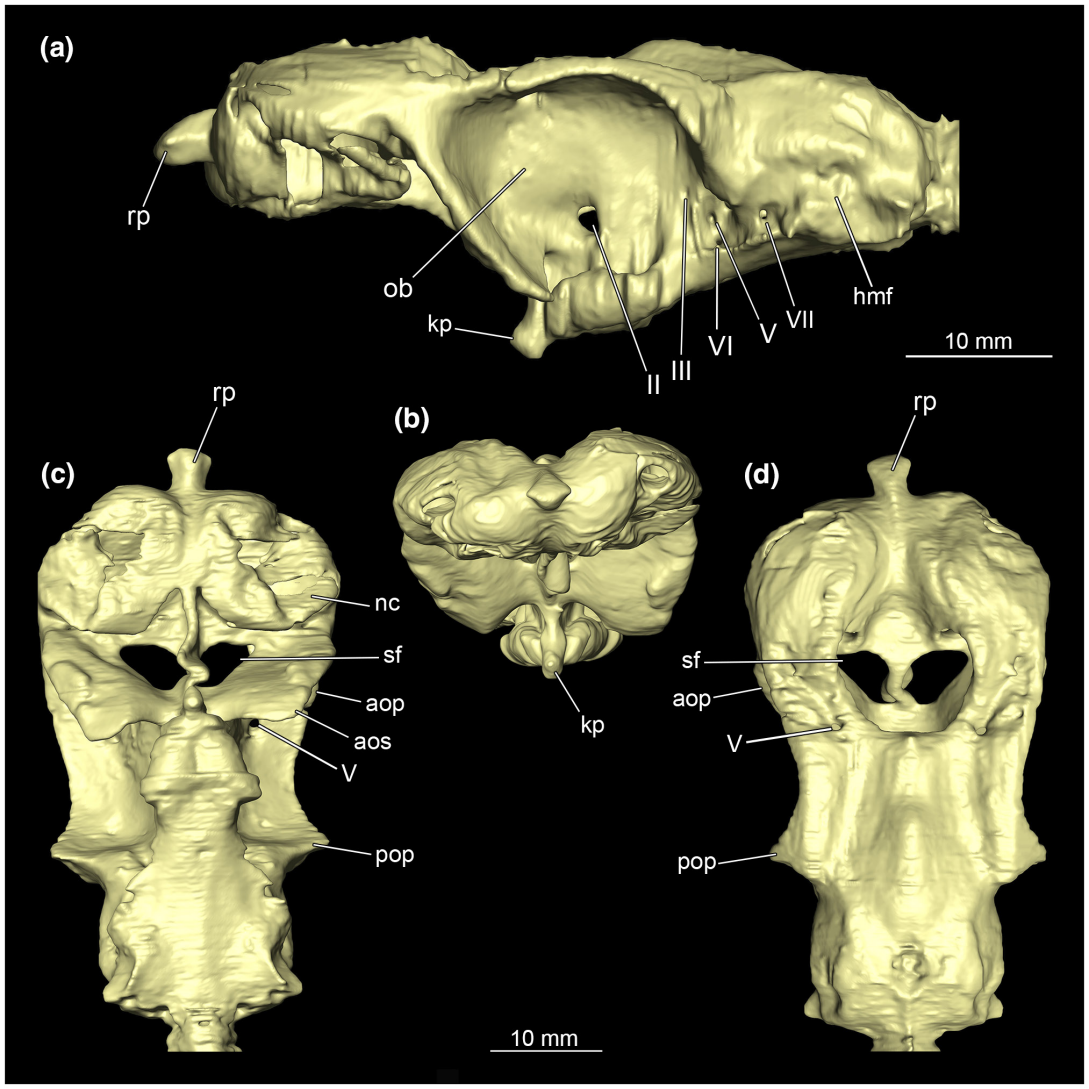
Chondrocranium of *Etmopterus lucifer*. (a) Lateral, (b) anterior, (c) ventral and (d) dorsal view of the chondrocranium. Abbreviations: II‐VII, fossa of the cranial nerve II‐VII; aos, antorbital shelf; aop, antorbital process; hmf, hyomandibular fossa; kp, keel process; nc, nasal capsule; ob, oribita; poc, preorbital canal; rp, rostral process; sf, subnasal fenestrae

The chondrocranium is a large single element forming a very distinctive structure that contains the brain and associated components. The nasal capsules are close to the preorbital wall, therefore the inter‐orbitonasal distance is relatively short. They are separated from each other by an internasal septum. The subnasal or rostral windows are clearly defined and kidney‐shaped (Figure 3c,d). They extend from the dorsal to the ventral at the posterior ends of the nasal capsules. Ventrally, they proceed between the preorbital wall and the nasal capsules. The anterior fontanelle of the ectethmoid chamber, characteristic of all Etmopteridae, (Shirai & Nakaya, 1990a, Figure 2d) is not discernable in our data (Figure 3). The orbital region contains a large eye socket occupying about a third of the total cranium (Figure 3a). The superficial ophthalmic nerve (cranial nerve V) perforates the cranium first at the posterior end of the eye socket, passes along the wall of the orbit and perforates again the chondrocranium at the posterior end of the nasal capsule and opens at the anterior part of the preorbital wall (Figure 3a,c,d). The eye stalk is very short and does not reach the eyeball. The dorsally located suborbital keel process (Figure 3a) appears broad and compact.

##### 3.1.2.1 Mandibular arch and dentition

**FIGURE 4:**
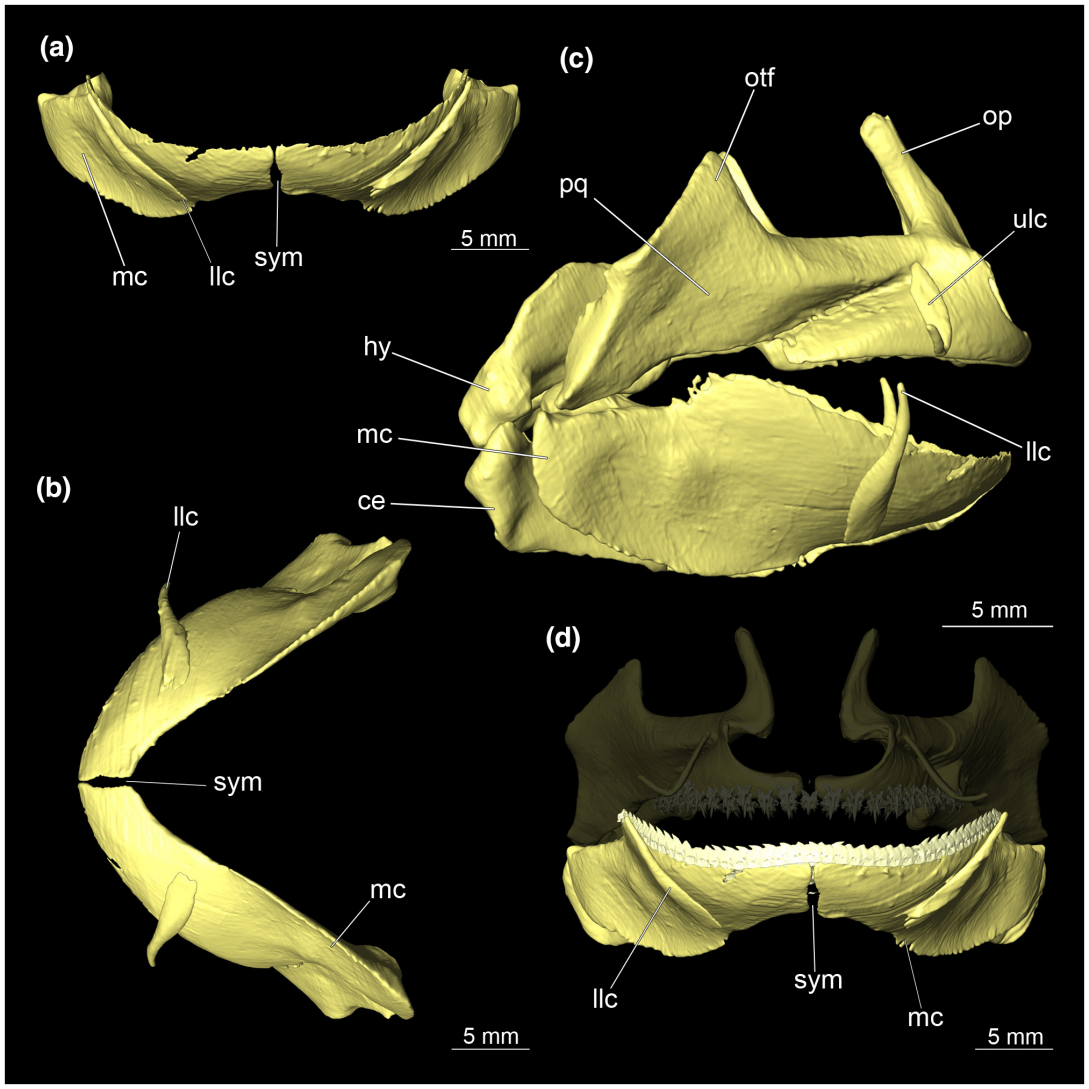
Meckel's cartilage, labial cartilage and complete mandibular arch of *Etmopterus lucifer*: (a) anterior view, (b) ventral view, (c) entire mandibular arch and parts of the hyoid arch lateral, (d) anterior view of the Meckel's cartilage with dentition and lower jaw labial cartilage. Abbreviations: ce, ceratohyale; hy, hyomandibula; llc, lower labial cartilage; mc, Meckel's cartilage; op, orbital process; otf, otic flange; pq, palatoquadratum; sym, symphysis; ulc, upper labial cartilage

**FIGURE 5:**
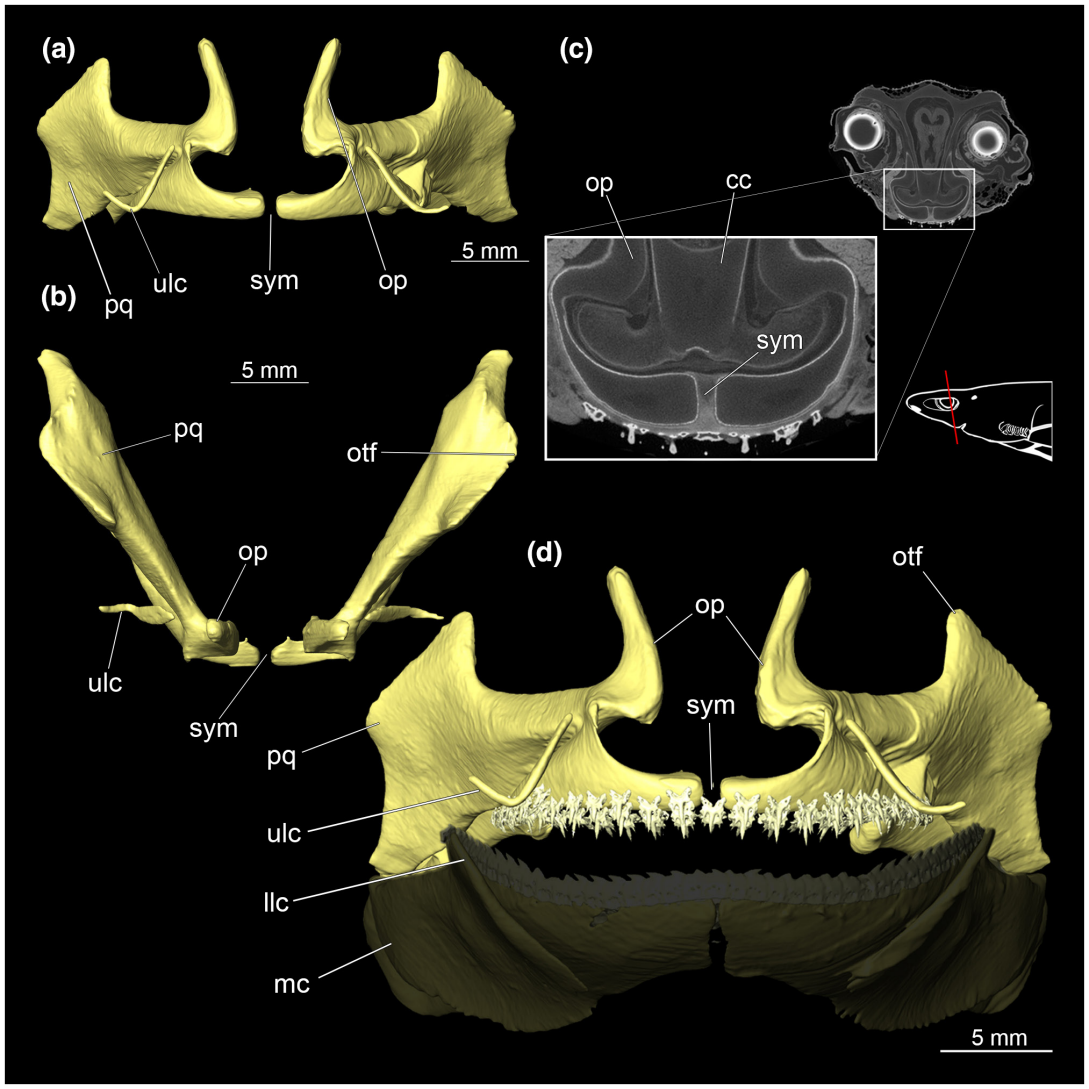
Palatoquadratum of *Etmopterus lucifer*: (a) anterior view, (b) dorsal view, (c) CT image shows the connection of both elements of the upper jaw via connective tissue, transversal, warped unstained (Etmop‐lucif‐ZSM‐30813) and 120 h stained (Etmop‐lucif‐ZSM‐30813_ai2mu) data sets, (d) anterior view of the palatoquadratum with dentition and upper labial cartilage, Meckel's cartilage with dentition displayed transparently. Abbreviations: llc, lower labial cartilage; mc, meckel's cartilage; op, orbital process; otf, otic flange; pq, palatoquadratum; sym, symphysis, ulc, upper labial cartilage

The mandibular arch is composed of the lower Meckel's cartilage and the palatoquadratum (Figures 4 and 5).

The palatoquadratum consists of two antimeres. Both cartilages are connected mesially by soft tissue along the symphysis (Figure 5c). In the middle, the palatoquadratum shows an orbital process that extends dorsally close to the symphysis (Figures 5a,c,d and 7d). From a lateral view, a process pointing dorsally protrudes at about half of the length of the palatoquadratum (Figure 4c).

**FIGURE 6:**
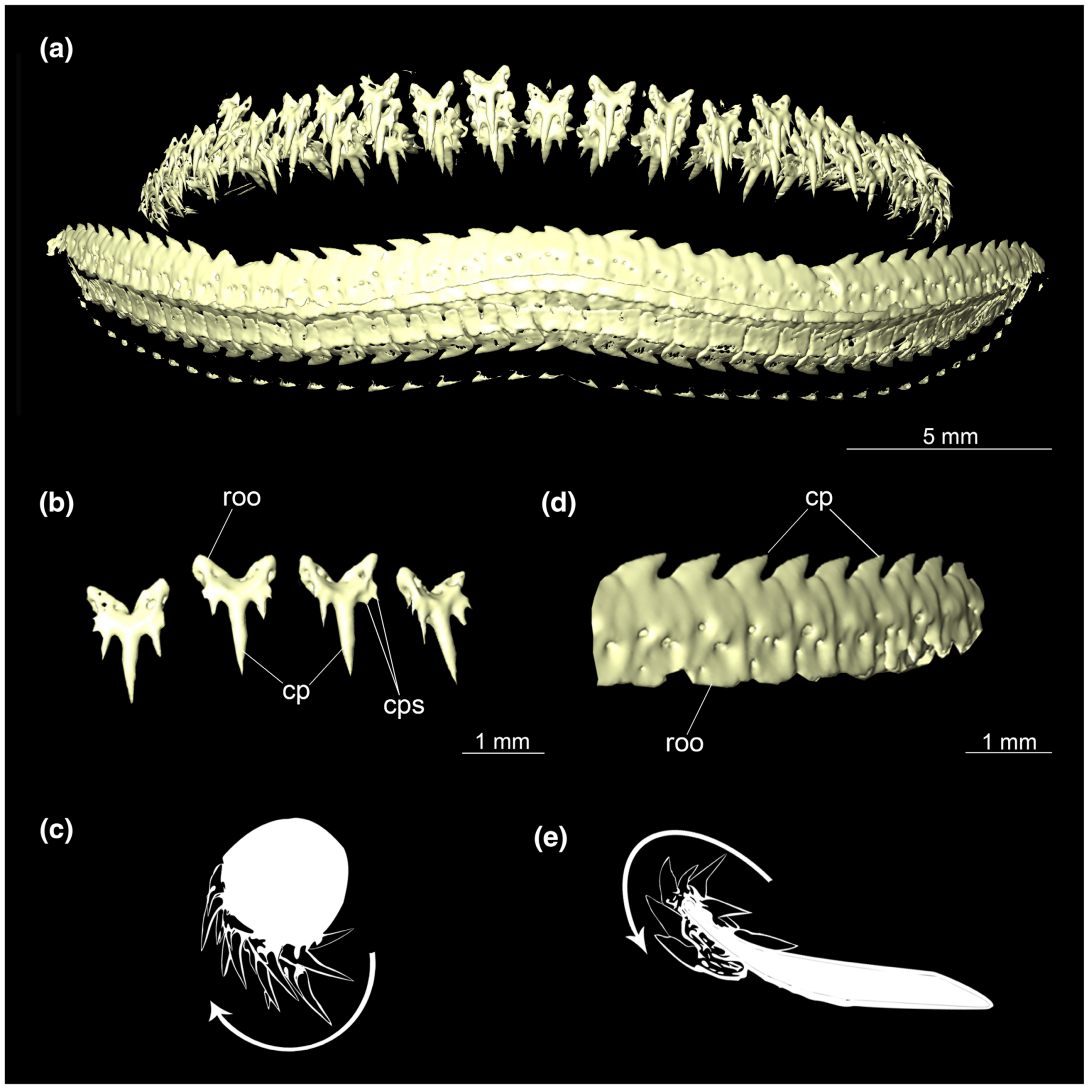
Dentition of *Etmopterus lucifer*: (a) upper jaw (top) and lower jaw (bottom) anterior, (b) teeth of the upper jaw anterior, (c) schematic cross‐section of the upper jaw, an arrow pointing in the direction of tooth replacement, (d) teeth of the lower jaw anterior, (e) schematic cross‐section of the lower jaw, arrow pointing in the direction of tooth replacement. Abbreviations: cp, cusp; cps, cusplets; roo, root

**FIGURE 7:**
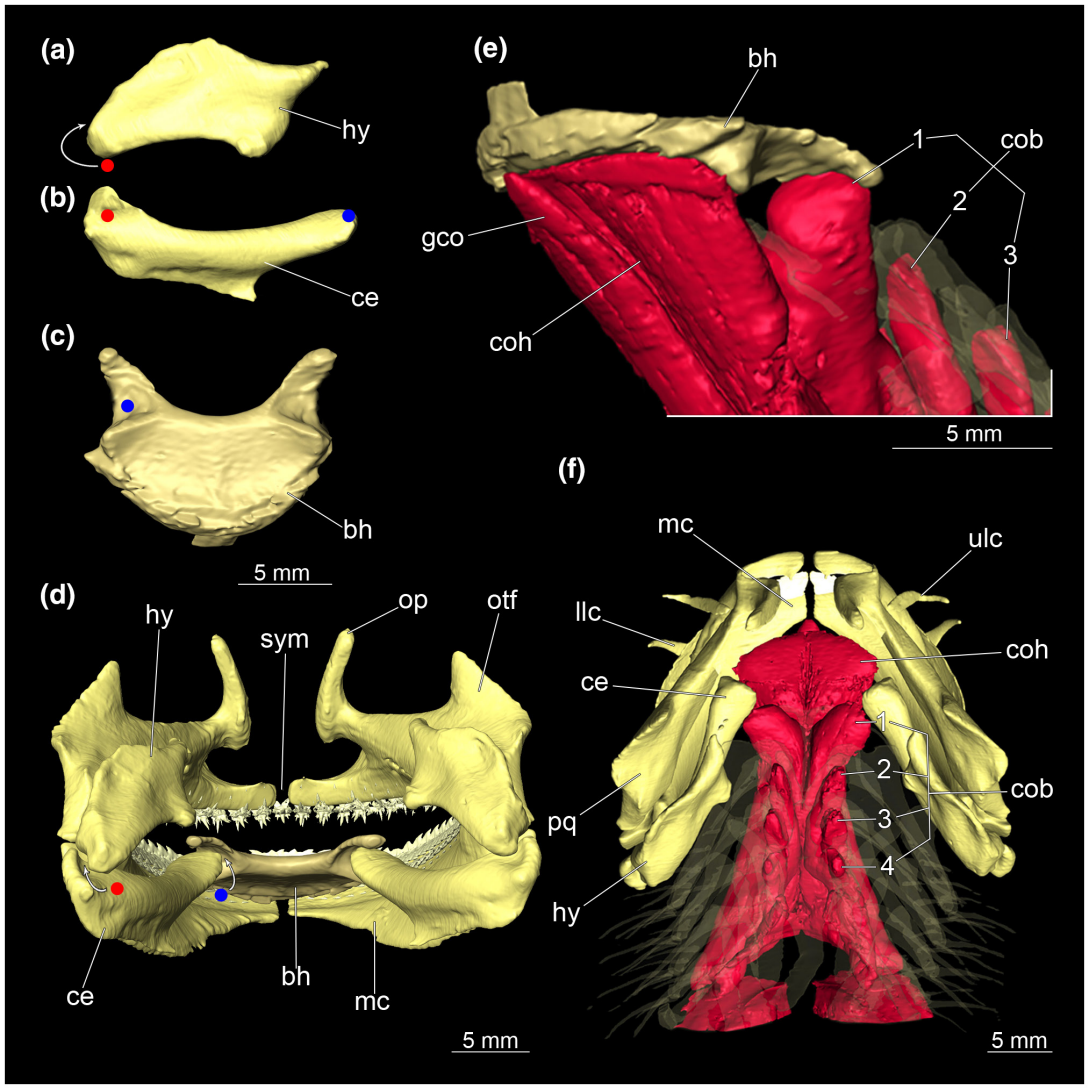
Hyoid arch and hypobranchial musculature of *Etmopterus lucifer*, the different coloured dots and arrows indicate areas where the individual elements of the hyoid arches interact with each other: (a) proximal lateral view of the hyomandibula, (b) proximal lateral view of the ceratohyale, (c) ventral view of the basihyale, (d) anteriat view of the mandibular and hyoid arch with dentition, (e) interaction of the basihyale with the hypobranchial muscles (branchial arches displayed transparently), (f) dorsal view of the mandibular, hyoid and branchial arches with the complete system of the hypobranchial muscles, whereby the basihyale was excluded and the branchial arches are displayed transparently for reasons of clarity. Abbreviations: bh, basihyale; ce, ceratohyale; cob; *Musculus coraco‐branchialis*; coh, *Musculus coraco‐hyoideus*, gco, *Musculus genio‐coracoideus*; hy, hyomandibula; llc, lower labial cartilage; mc, Meckel's cartilage; op, orbital process; otf, otic flange; pq, palatoquadratum; sym, symphysis; ulc, upper labial cartilage

The dentition of the upper jaw extends from the central symphysis to about the level of the base of the optic flange. The teeth are arranged in rows and files. Following Jambura et al. (2018), tooth rows extend from mesial to distal and tooth files from labial to lingual (Figure 6a–c). No distinct symphyseal teeth are present (Figures 5d and 7d).

Each tooth consists of a slender pointed main cusp, which is laterally accompanied at the base by two to four cusplets (Figure 6b). The three to four functional rows of teeth are located at the outer margin of the upper jaw. The replacement teeth start developing at the lingual side of the palatoquadrate with the tip pointing dorsally and turning ventrally over time when the previous teeth are worn (Figure 6c). Several foramina are visible on the root (Figure 6b).

The Meckel's cartilage appears considerably more planar than the palatoquadratum. (Figure 4). The lower jaw is composed of two separate elements, connected centrally along the symphysis by soft tissue. The caudal end of Meckel's cartilage is pointing outwards forming a groove. Dorsally to this groove lies the quadratomandibular joint which is the articulation of the palatoquadratum and the Meckel's cartilage.

The teeth of the lower jaw differ fundamentally in morphology from those of the upper jaw; the base appears flat and rectangular. The cusp is bent laterally and forms a cutting edge (Figure 6a,d). The cutting edge has no serration. The teeth are overlappingly arranged and are connected by connective tissue (Figure 6d), which supports the replacement of complete tooth rows in the lower jaws of *Etmopterus* (Figure 6e).

A pair of labial cartilages support the upper and lower lips. The lower labial cartilage is lying lateral at the 10th tooth counting from the symphysis. It protrudes significantly beyond the dorsal mandibular rim (Figure 4c). The upper labial cartilages are situated between the 6th and 7th upper jaw teeth. Compared to the lower labial cartilage, they lie more anteriorly and are curved pointing laterally (Figure 5a,b,d).

##### 3.1.2.2 Hyoid arch

The hyoid arch consists of five individual elements: One central unpaired basihyale, paired ceratohyale and paired hyomandibulae (see Figure 7). The shape of the hyomandibula reminds roughly of a parallelogram and is laterally flattened (Figure 7a). The posterior part of the hyomandibula interacts with the posterior part of the ceratohyale (Figure 7a,b,d). The ceratohyale is about 20% longer in size compared to the hyomandibula (Figure 7b). It is generally more elongated than the hyomandibula and interacts at the anterior end with the basihyale (Figure 7a,b,d). The latter is located on the bottom of the oral cavity and is posteriorly connected to the ceratohyals through two grooves on the dorsal side (Figure 7c). These grooves have a socket‐like appearance. The upper areas are elongated and have relatively pointed extensions in the posterior direction (Figure 7d). The anterior part of the basihyal is semicircular and lies on top of the *Musculus coraco‐hyoideus* (Figure 7e,f).

##### 3.1.2.3 Branchial arches

**FIGURE 8:**
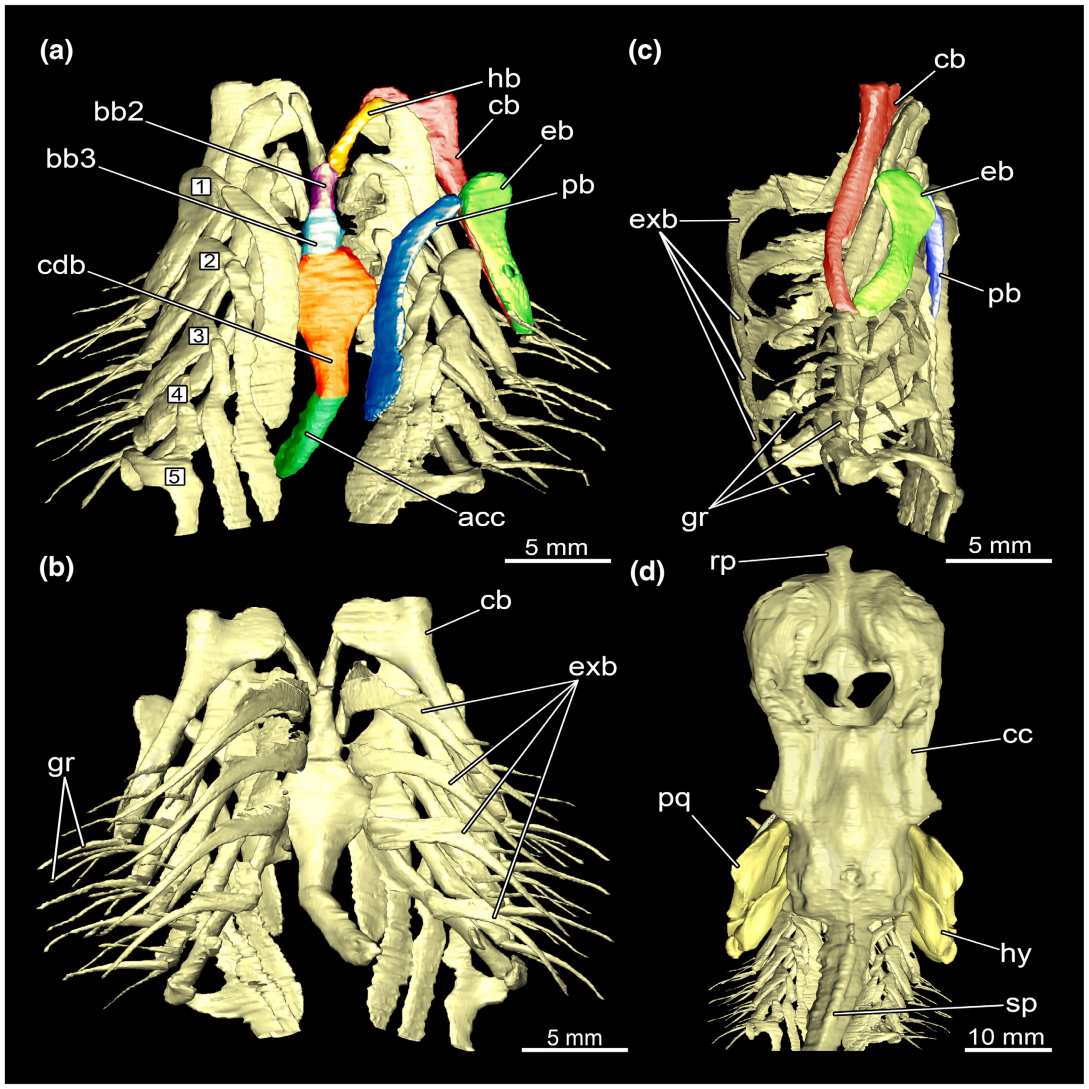
Branchial arches of *Etmopterus lucifer*, numbers in boxes indicate the single arches: (a) ventral view with individual elements labelled in different colours, (b) dorsal view, (c) lateral view with individual elements labelled in different colours, (d) dorsal view of the chondrocranium in the system with the mandibular arch, the hyoid arch and the branchial arches. Abbreviations: acc, accessory cartilage of basibranchiale; bb (2/3), basibranchiale; cb, ceratobranchiale; cc, chondrocranium; cdb, cardiobranchiale; eb, epibranchiale; exb, extrabranchial cartilage on branchial arches; gr, gill ray; hb, hypobranchiale; hy, hyomandibulare; pb, pharyngobranchiale; pq, palatoquadratum; rp, rostral process; sp, spine

The branchial arches (see Figure 8) are located Comment on behind the hyoid arch. The branchial arches of *E. lucifer* consist of five pairs of arches. Each individual arch consists of the following cartilages: pharyngobranchiale, epibranchiale, ceratobranchiale, hypobranchiale and basibranchiale (Figure 8a,c).

The pharyngobranchiale is the most dorsal cartilaginous element. At the caudal end, it connects to the vertebral column. From there, it protrudes cranially in an outward‐extending arch. Anteriorly, the pharyngobranchiale articulates with the epibranchiale. The epibranchiale is shorter and stouter. It extends from anterior to posterior. The cranial end is broader and shovel‐like. At about two thirds of the length, it shows a large central foramen for the branchial nerve branches. At the caudal end, it connects to the ceratobranchiale. The ceratobranchiale is narrow posteriorly, getting broader at the anterior end and tapering off into a paddle‐shaped widening articulating with the epibranchiale. This element is elongated and rather narrow and gets reduced in length in every arch. It is missing completely in the last two branchial arches. It extends roughly in a craniocaudal direction and articulates anteriorly with the ceratobranchiale and posteriorly with the basibranchiale. The basibranchiale is an unpaired element located dorsal of the cardial region (Shirai, 1992b). It is composed of a single row of at least partially fused cartilages. Basibranchiale 2 and 3 form the anterior basibranchiale. Posteriorly of these elements sits a pair of large single elements, the cardiobranchiales; these are much wider than the two anterior elements. The most posterior part is called the accessory cartilage. This structure appears rather slim and elongated. In addition, the dorsal and ventral extrabranchial cartilage can be found (Figure 8b,c). The upper part of the extrabranchial cartilage cannot be discerned in our data. Between four and six gill rays are connected laterally to each branchial arch (Figure 8a–c).

### 3.2 Soft tissues

#### 3.2.1 Muscles

**FIGURE 9:**
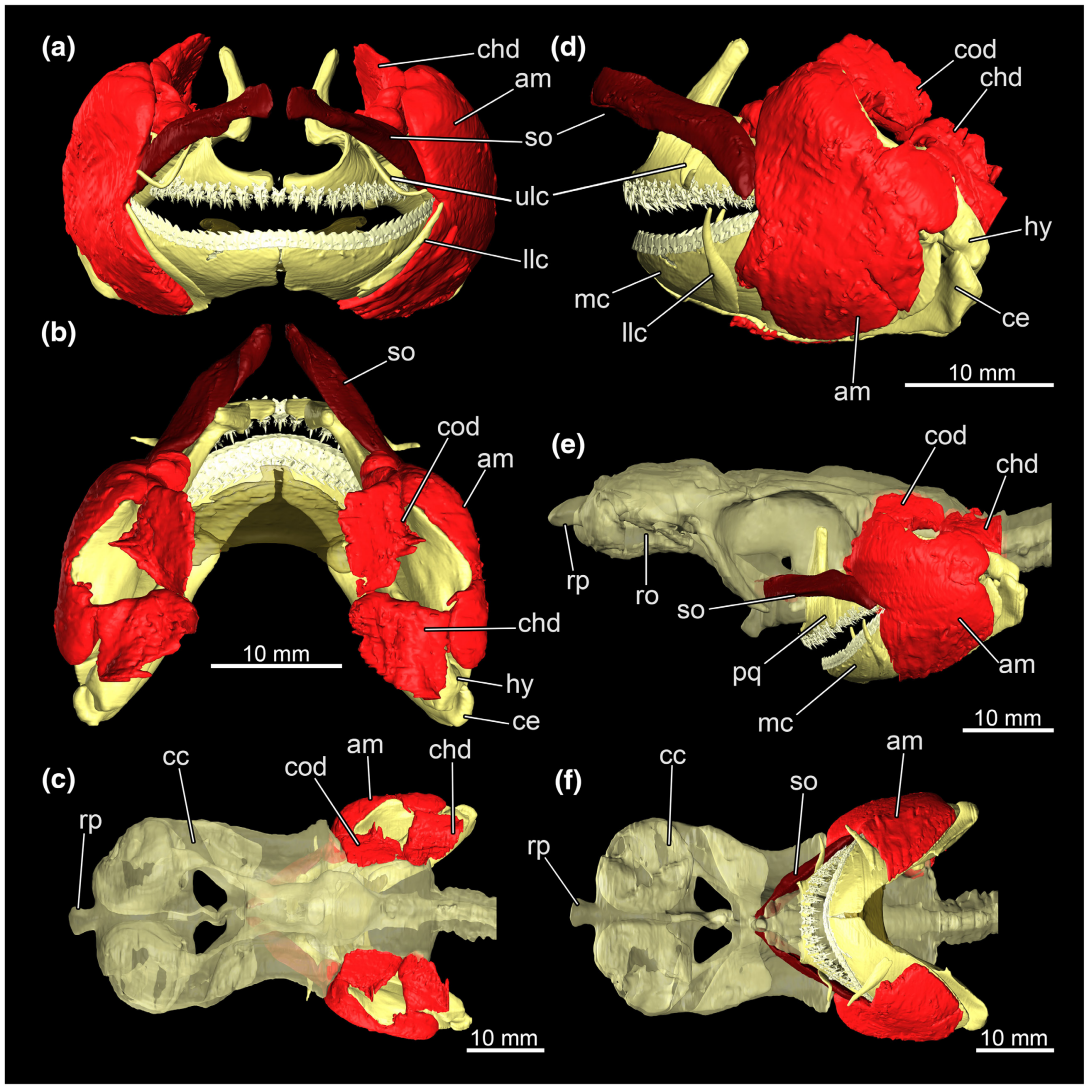
Muscles of the jaw apparatus of *Etmopterus lucifer*: (a) anterior view of the jaws with the involved muscle groups, (b) dorsal view of the jaws with the involved muscle groups, (c) dorsal view of the chondrocranium with the jaws apparatus and the jaw muscles, (d) lateral view of the jaws with the involved muscle groups, (e) lateral view of the chondrocranium with the jaws apparatus and the jaw muscles, (f) ventral view of the chondrocranium with the jaws apparatus and the jaw muscles. Abbreviations: am, *Musculus adductor mandibulae*; ce, ceratohyale; chd; *Musculus constrictor hyoideus dorsalis*; cod, *Musculus constrictor dorsalis*; hy, hyomandibula; llc, lower labial cartilage; mc, Meckel's cartilage; pq, palatoquadratum; ro, rostrum; rp, rostral process; so, *Musculus suborbitalis*; ulc, upper labial cartilage

The posterior half of the jaw is laterally surrounded by the massive *Musculus adductor mandibulae*. It attaches to the palatoquadrate and extends ventrally to the Meckel's cartilage (Figure 9a–f). Starting from the base of the keel process, a muscle bundle runs parallel to the orbit towards the posterior end of the mouth opening, to the *M. suborbitalis* (Figure 9a,b,d–f). The *M. adductor mandibulae superficialis* could not be found. The *M. constrictor dorsalis* inserts at the dorsal part of the palatoquadratum. It extends from the dorsal edge of the palatoquadratum anteriorly from the orbit posteriorly to the anterior wall of the spiracle (Figure 9b–e).

**FIGURE 10:**
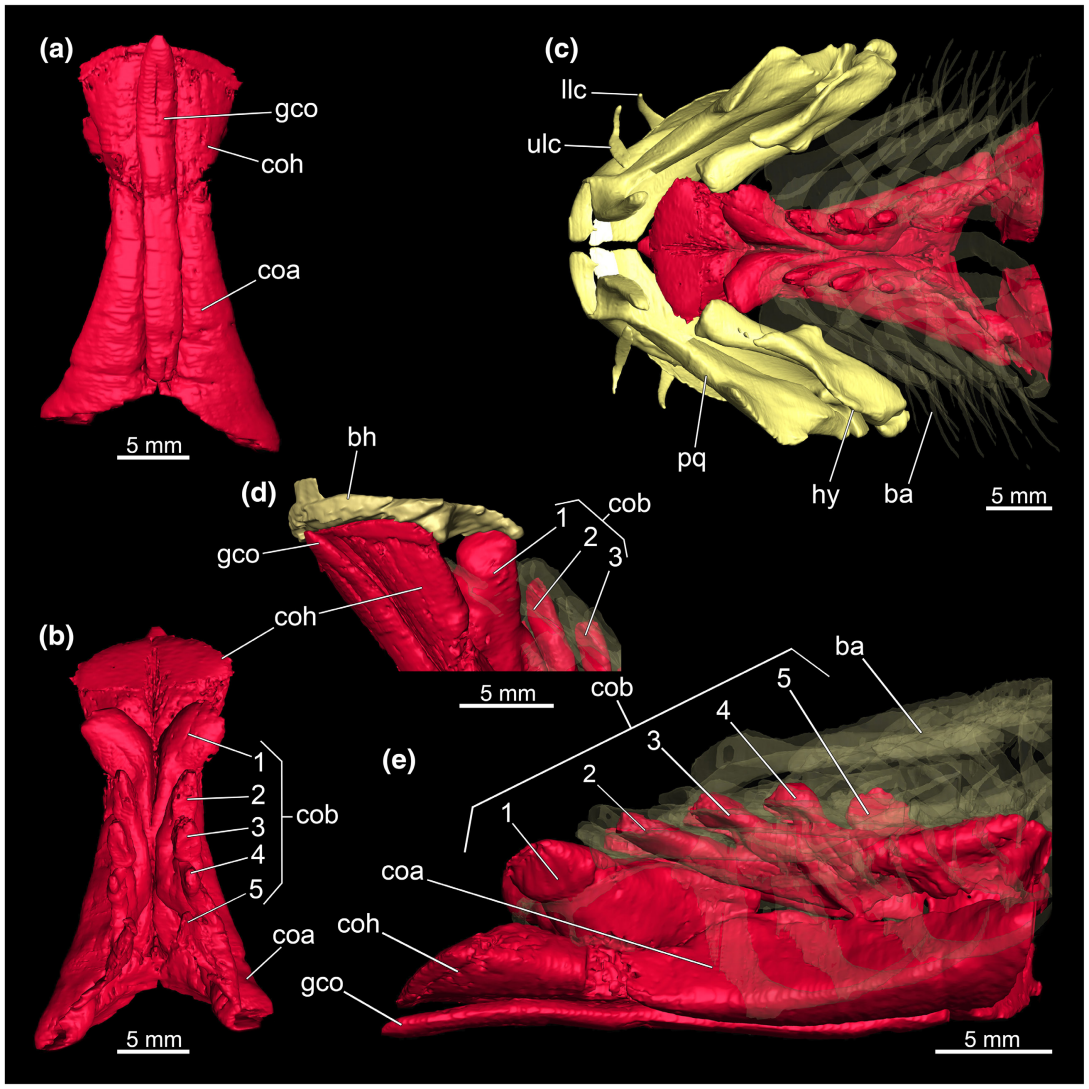
Hypobranchial musculature of the *Etmopterus lucifer* (a) dorsal view of the muscle group, (b) ventral view, (c) dorsal view of the mandibular arch and the hyoid arch with the complete system of the hypobranchial muscles, the basihyale was excluded and the branchial arches are displayed transparently for reasons of clarity, (d) antero‐lateral view of the basihyale with the involved muscle group, (e) lateral view of the Hypobranchial musculature. Abbreviations: ba, branchial arches; bh, basihyale; coa, *Musculus coraco‐arcualis*, cob; *Musculus coraco‐branchialis*; coh, *Musculus coraco‐hyoideus*, gco, *Musculus genio‐coracoideus*; hy, hyomandibula; llc, lower labial cartilage; pq, palatoquadratum; ulc, upper labial cartilage

The hypobranchial muscles are prominent and well recognizable (Figure 10) located ventrally below the branchial arches. The muscles contained within this muscle group are the *M. genio‐coracoideus*, the *M. rectus‐cervicis* and the *M. coraco‐branchialis*. The *M. genio‐coraoideus* extends from a fascia of the *M. rectus‐cervicis* ventral to the symphysis of the Meckel's cartilage. It appears thin, long and tapers anteriorly as well as posteriorly towards a rounded end (Figure 10a,d,e). The *M. rectus‐cervicis* runs parallel to the *M. genio‐coracoideus*. It is composed of a pair of parallel muscle bundles. Reaching about one‐third of the anterior part of the *M. rectus‐cervicis*, the muscle bundles are separated from the posterior part by a septum. The anterior part, the so‐called *M. coraco‐hyoideus*, inserts at the ventral side of the basihyale. The posterior part is called *M. coraco‐arcualis* and originates from the antero‐ventral part of the coracoid (Figure 10a,b,e). The five *M. coraco‐branchiales* above the *M. rectus‐cervicis* vary in size from posterior to anterior. The rearmost strand appears significantly smaller than the four front parts and the foremost strand appears thicker in comparison to the four rear strands. The three median strands have almost the same size (Figure 10f,d,e). The first strand of the *M. coraco‐branchiales*, along with the *M. genio‐coracoideus* emerges from a fascia of the *M. rectus‐cervicis*, called M*. coraco‐hyomandibularis*. It connects to the hyoid arch, more specifically to the posterior protrusions of the basihyale (Figure 10a–e). The four posterior strands of the *M. coraco‐branchialis* originate from the anterior part of the coracoid and insert in the *M. hypobranchialis* or the *M. cardiobranchialis* (Shirai, 1992b).

#### 3.2.2 Brain and nervous system

**FIGURE 11:**
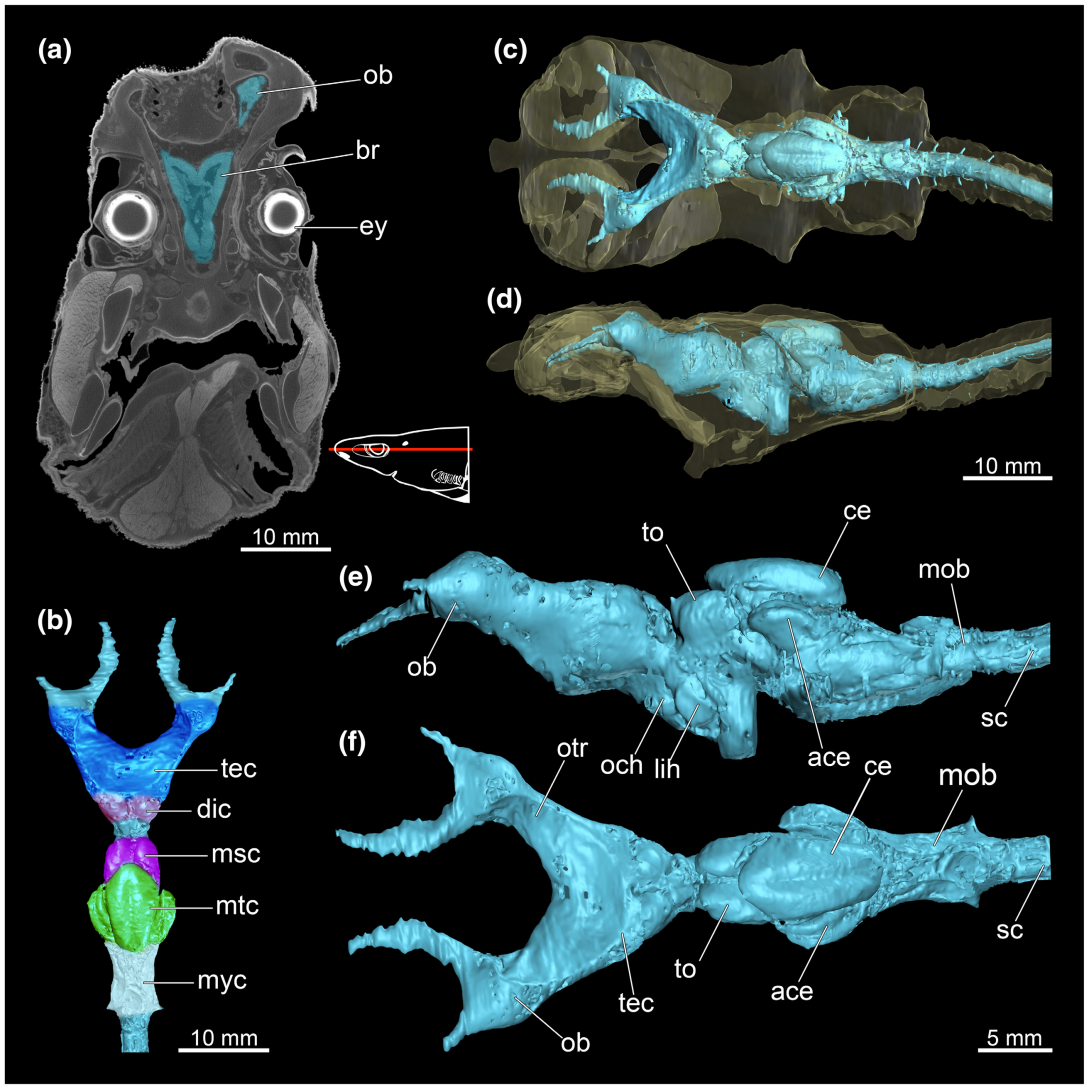
Brain of *Etmopterus lucifer*, showing the different areas of this part of the central nervous system. (a) Single image of the CT scan shows the structure of the brain in a cross‐sectional view, coronal, warped unstained (Etmop‐lucif‐ZSM‐30813) and 120 h stained (Etmop‐lucif‐ZSM‐30813_ai2mu) data sets; the red line in the scheme indicates the plane from which the slice originates in the CT scan, (b) dorsal view of the brain, showing the individual parts of the brain, (c) dorsal view of the brain which illustrates the position within the chondrocranium (latter displayed translucently), (d) lateral view of the brain which illustrates the position within the chondrocranium (latter displayed translucently), (e) lateral view of the brain showing the single lobes and areas, (f) dorsal view of the brain showing the single lobes and areas. Abbreviations: ace, auricle of cerebellum; br, brain; ce, cerebellum; dic, diencephalon; ey, eye; lih, lobus inferior hypothalami; mob, medulla oblongata; msc, mesencephalon; mtc, metencephalon; myc, myelencephalon; och, optical chiasma; ob, olfactory bulbs; otr, olfactory trunk, sc, spinal cord; tec, telencephalon; to, tectum opticum

Our CT data allow identification of individual brain components, that are olfactory bulbs, telencephalon, diencephalon, mesencephalon, metencephalon (cerebellum) and myelencephalon (medulla oblongata) (Figure 11b). The foremost region comprises the olphactoric bulbs and the olphactoric tracts, leading into the largest region of the forebrain, called telencephalon. It leads into the cerebral hemispheres (Figure 11e,f). In their entirety, these brain components form the cerebrum. The diencephalon is located posteriorly to the telencephalon. It contains multiple subdivisions, for example the epithalamus, the thalamus and the hypothalamus. According to Yopak (2022), the function of the diencephalon in cartilaginous fishes is still not entirely clarified. The author suggests that it is a ‘multimodal relay centre’ (Yopak, 2022). Further, it seems to regulate a variety of homeostatic functions (e.g. feeding and reproduction). The roof of the mesencephalon comprises the two prominent lobes of the optic tectum (Figure 11e,f). The optic tectum is most likely associated with the sense of sight and processes visual stimuli input. The lobus inferioris hypothalami and the optical chiasma are located below the optic tectum, where the optic nerves originate (Figure 11e,f). Dorsal to the mesencephalon the hindbrain is situated comprising both the cerebellum and the medulla oblongata. The cerebellum represents the dorsal‐most part of the brain (Figure 10e,f). Along with the auricles of the cerebellum (Figure 11e,f), the cerebellum forms the metencephalon. The cerebellum of *E. lucifer* shows a low degree of foliation; Yopak and Montgomery (2008) gave it a foliation index of 1. The most posterior area in the brain of *E. lucifer* is the myelencephalon. The main structure of the myelencephalon is the medulla oblongata, which merges further backwards into the spinal cord (Figure 11e,f).

**FIGURE 12:**
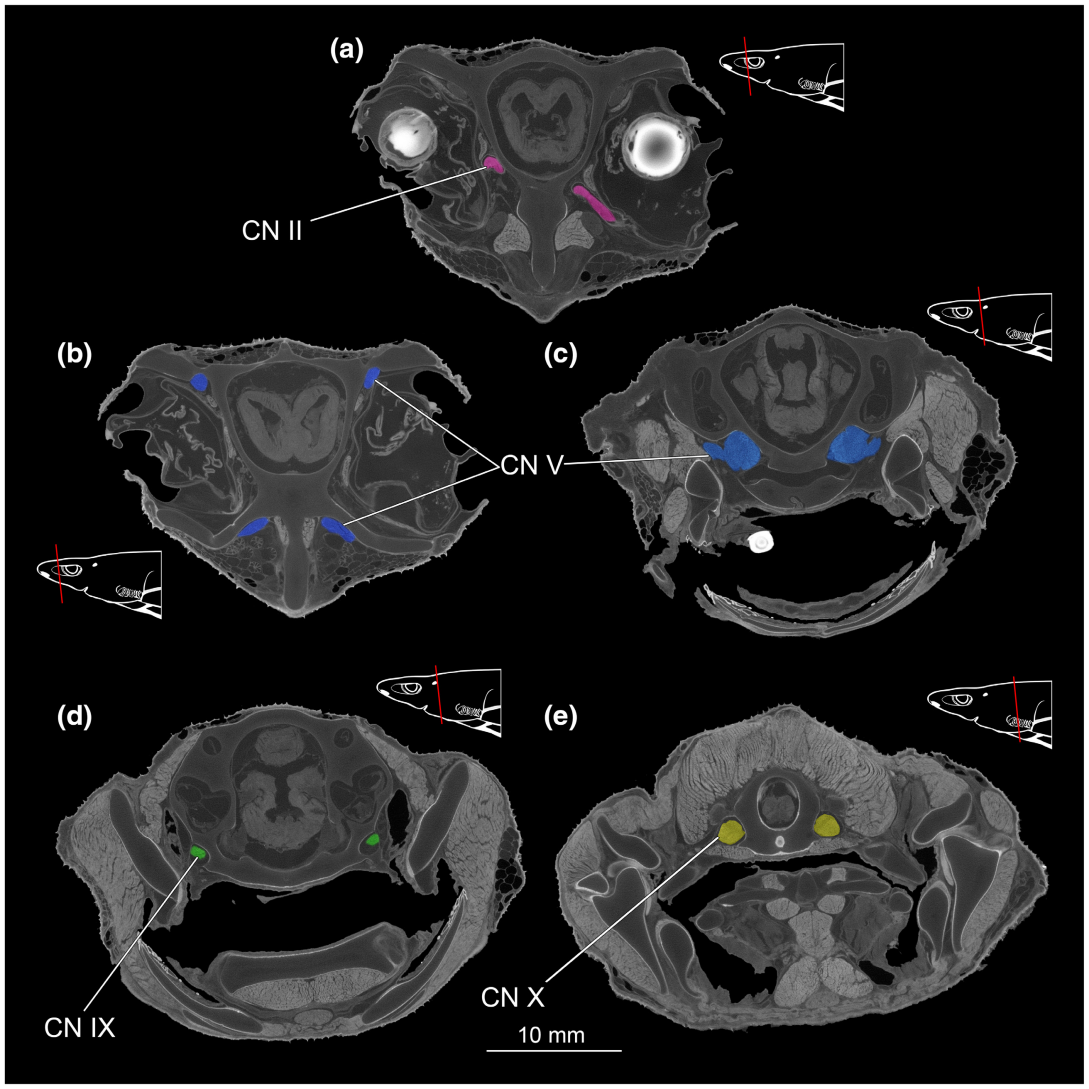
CT images of *Etmopterus lucifer* show different areas of the cranium with the different nerves observable in the cross‐section, warped unstained (Etmop‐lucif‐ZSM‐30813) and 120 h stained (Etmop‐lucif‐ZSM‐30813_ai2mu) data sets. Nerves were colourised for better visibility. The red line in the scheme indicates the plane from which the single image originates within the CT scan; (a) CT image shows the cranial nerve II (*Nervus opticus*) in pink, transversal cut, (b) the dorsal and the ventral branches of the cranial nerve V (*Nervus trigeminus*) in blue in the anterior region of the head, transversal cut, (c) the transition of the cranial nerve V (*Nervus trigeminus*) into the chondrocranium and later into the brain in the slightly further back region of the head, marked in blue, transversal cut, (d) the transition of the cranial nerve IX (*Nervus glossopharyngeus*) into the chondrocranium, marked in green, transversal cut, (e) the transition of the cranial nerve X (*Nervus vagus*) into the chondrocranium, marked in a yellow, transversal cut. Abbreviations: CN II, cranial nerve II (*Nervus opticus*); CN V, cranial nerve V (*Nervus trigeminus*); CN IV, cranial nerve IX (*Nervus glossopharyngeus*); CN X, cranial nerve X (*Nervus vagus*)

Various nerve cords, such as the cranial nerves II (*Nervus opticus*; Figure 12a), the cranial nerves V (*N. trigeminus*) (Figure 12b,c), the cranial nerves IX (*N. glossopharyngeus*) (Figure 12d) and the cranial nerves X (*N. vagus*) (Figure 12e) are clearly discernible in the CT data.

#### 3.2.3 Nose

**FIGURE 13:**
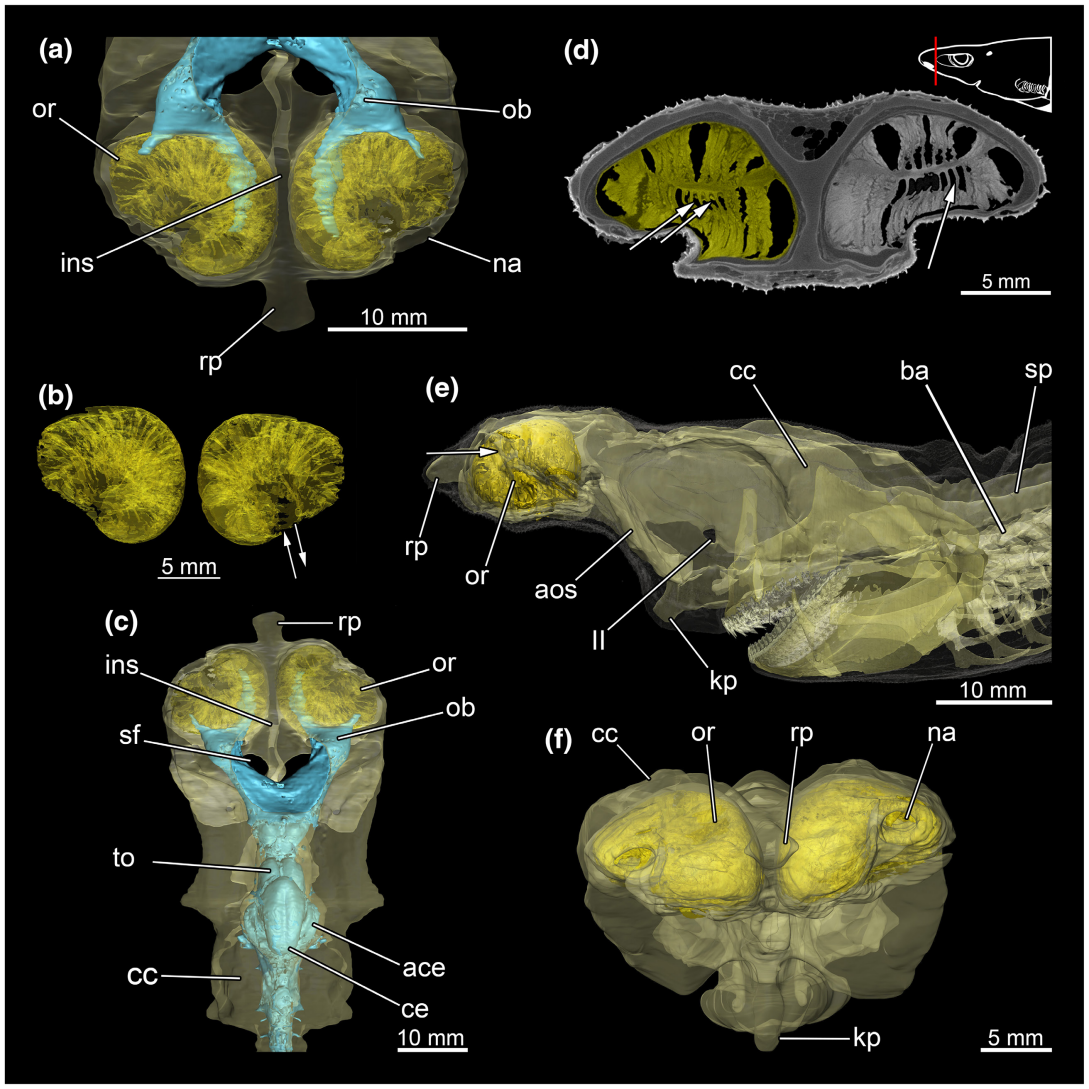
Olfactory sensory organ of *Etmopterus lucifer*, showing the distinct nasal capsules, the olfactory rosettes containing the olfactory epithelium and associated systems. (a) Dorsal view of the olfactory rosettes within the chondrocranium and the olfactory trunks subsequent to the olfactory bulbs; (b) isolated olfactory rosettes with recognizable folding within the epithelium (lamellae), arrows show the in‐ and outflow of the water; (c) dorsal view of the chondrocranium with the olfactory rosettes, the olfactory bulbs and the olfactory trunks within the chondrocranium; (d) CT image shows a cross‐section through the nasal capsules and the olfactory rosettes, arrows pointing out the olfactory lamellae, transversal cut, warped unstained (Etmop‐lucif‐ZSM‐30813) and 120 h stained (Etmop‐lucif‐ZSM‐30813_ai2mu) data sets; the red line in the scheme indicates the plane from which the single image originates in the CT scan; (e) lateral view of the chondrocranium, the mandibular arch, the branchial arches and the olfactory rosettes within the chondrocranium, arrow pointing out the naris; (f) anterior view of the chondrocranium and the olfactory rosettes. Abbreviations: II, fossa of the cranial nerve II; ace, auricle of cerebellum; aos, antorbital shelf; ba, branchial arches; cc, chondrocranium; ce, cerebellum; ins, internasal septum; kp, keel process; na, naris; ob, olfactory bulb; or, olfactory rosette; rp, rostral process; sf, subnasal fenestrae; sp, spine; to, tectum opticum

The olfactory rosettes lie in the nasal capsules that connect anteriorly to the orbits (Figure 13e). The individual olfactory rosettes appear kidney‐shaped with the inner cavity connected to the nostrils (Figure 13a,b,e,f). The epithelium as such is folded for surface enlargement and forms the so‐called olfactory lamellae (Figure 13d). The lamellae are connected to the olfactory bulbs, which transmit the stimuli of the sensory receptors via the olfactory tracts to the telencephalon (Figure 13a,c). The nasal capsule as well as the olfactory rosettes have no connection to the oral cavity.

#### 3.2.4 Eyes

**FIGURE 14:**
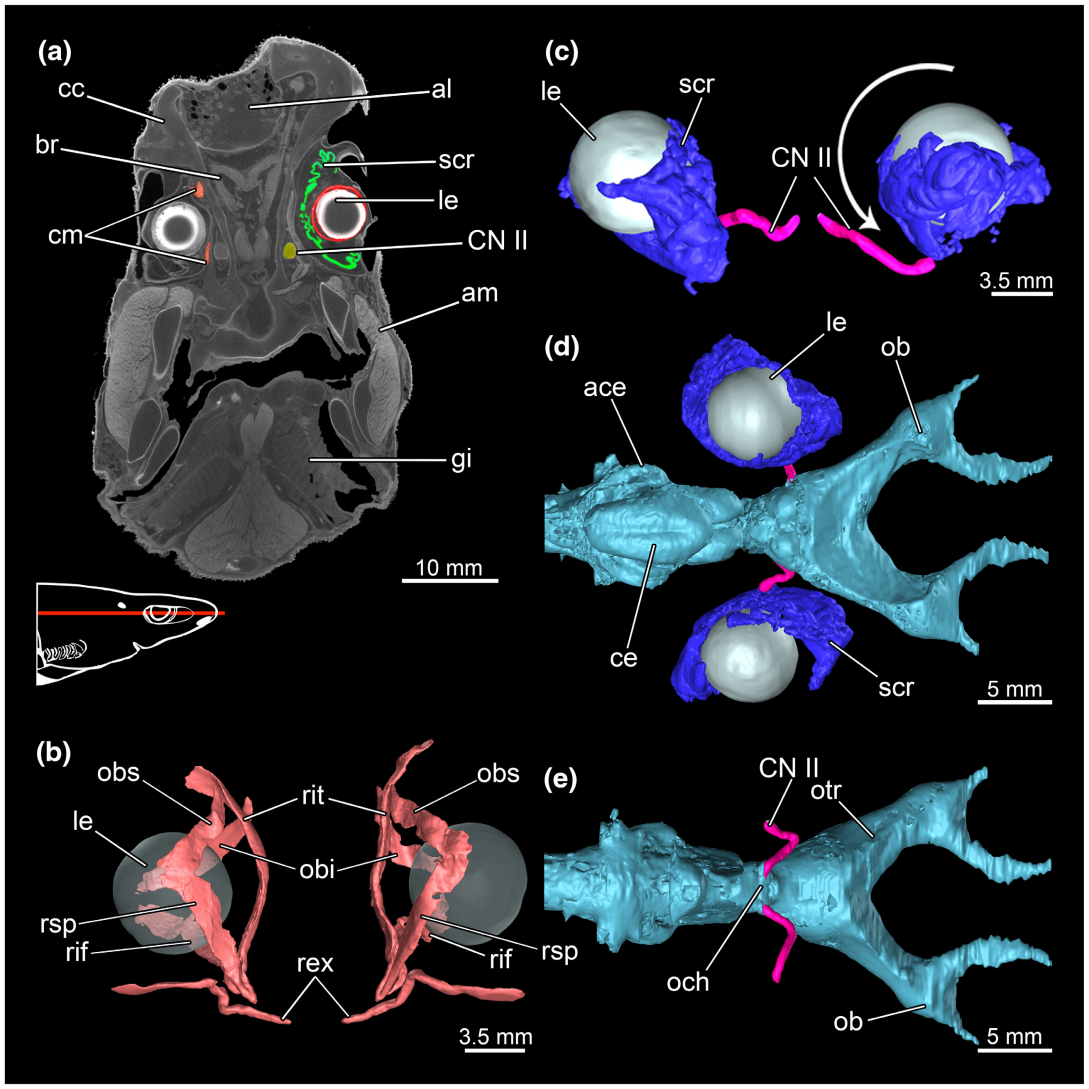
Involved organ systems of the sense of sight, including the nerves and ciliary muscles of *Etmopterus lucifer*. (a) CT image shows the colour labelled the *Nervus opticus* (yellow), the sensory epithel (green), the eyeball with lens (red) and the ciliary muscles (orange); the red line in the scheme indicates the plane from which the single image originates in the CT scan, coronal cut, warped unstained (Etmop‐lucif‐ZSM‐30813) and 120 h stained (Etmop‐lucif‐ZSM‐30813_ai2mu) data sets; (b) ciliary muscles, dorsal view (c) lens, sclera, cornea, choroid and suprachoroidea with *Nervus opticus*, anterior view, the arrow points out the direction of rotation around which the entire right structure is twisted, (d) lens, sclera, cornea, choroid and suprachoroidea, *Nervus opticus* and brain dorsal, (e) *Nervus opticus* with the transition to the brain at the optical chiasma, ventral view. Abbreviations: ace, auricle of cerebellum; al, ampullae of Lorenzini; am, adductor mandibulae; br, brain; cc, chondrocranium; ce, cerebellum; cm, ciliary muscles; CN II, cranial nerve II (*Nervus opticus*); gi, gill; le, lens; ob, olfactory bulb; och, optic chiasma; otr, olfactory trunk; obi, *Musculus obliquus inferioris*; obs, *Musculus obliquus superioris*; rex, *Musculus rectus externus*; rif, *Musculus rectus inferioris*; rit, *Musculus rectus internus*; rsp, *Musculus rectus superioris*; scr, sclera, choroid and retina

The lenses appear as a bright, well‐defined structure in the CT slices (Figure 14a). Their surface appears to be even and not affected by shrinkage. There is a strongly twisted and folded structure surrounding the eyeballs. This presumably is composed of the sclera (incl. scleral cartilage), the choroid (incl. tapetum lucidum) and the retina (Figure 14a,c,d). This tissue complex behind/below the left eye seems to be misplaced in comparison to the one of the right eye. The left one slid downwards and became located on the ventral half of the lens, while the right one lies from a lateral point of view behind the lens (Figure 14d). This structure serves as a point of contact for the optic nerve (CN II). The left eye's optic nerve slid downwards along with the connected folded tissue. The original curvature appears stretched (Figure 14c–e). The optic nerves originate from the optic chiasma in the mesencephalon (Figure 14e). There are two obliques and four rectus muscles. The *Musculus rectus superioris*, the *M. rectus internus* and the *M. rectus inferioris* originate at the base of the eye stalk in front of the *M. rectus externus* which emerges from a separate fossa (Figure 14b). The *Musculus obliquus superioris* and the *Musculus obliquus inferioris* arise from the preorbital wall; they are clearly separated from each other.

##### 3.2.4.1 Ampullae of Lorenzini

**FIGURE 15:**
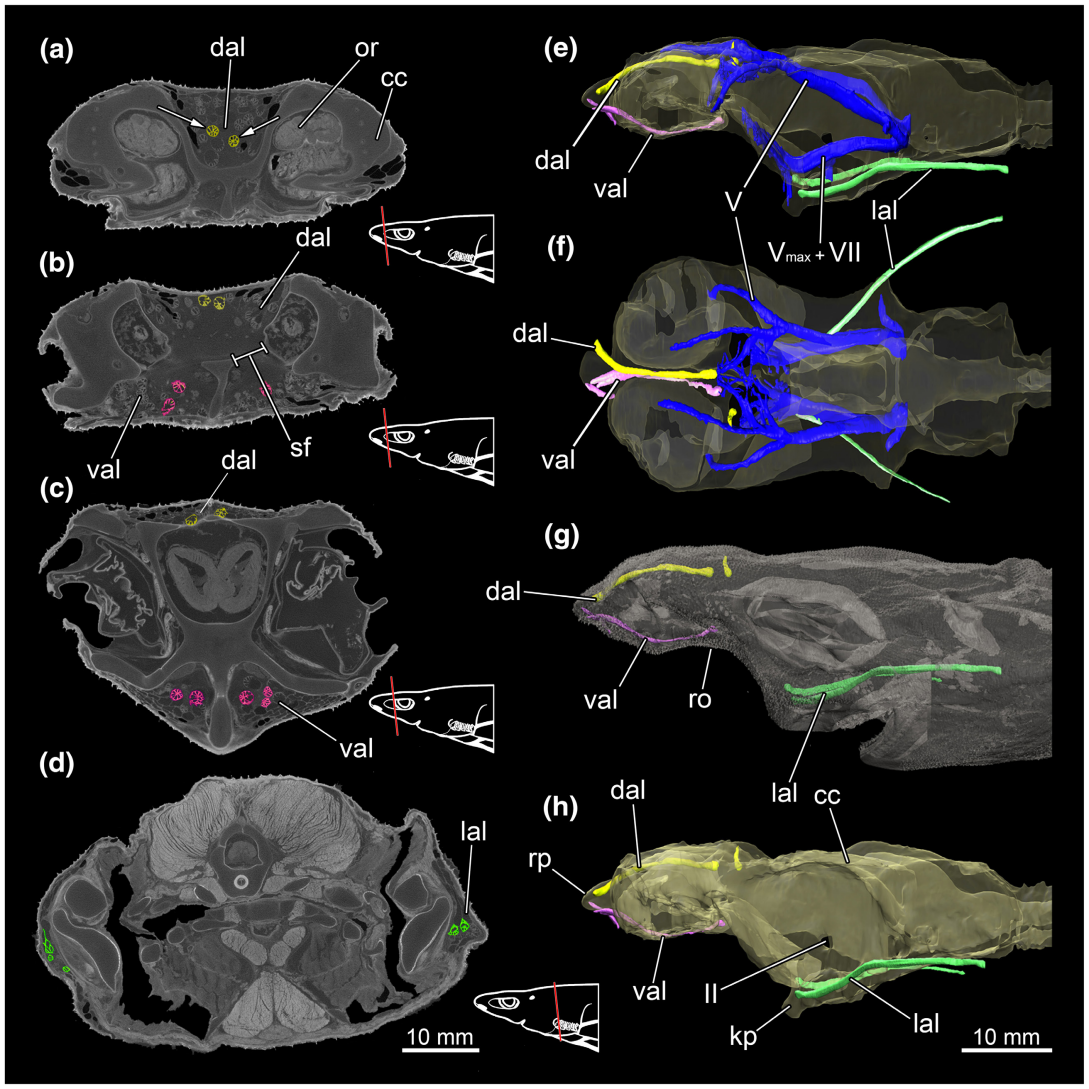
CT images and 3D reconstructions of *Etmopterus lucifer* show the different groups of ampullae of Lorenzini. For each area, a few ampullae were representatively colour‐coded (colour corresponds to the used colour in the 3D model), warped unstained (Etmop‐lucif‐ZSM‐30813) and 120 h stained (Etmop‐lucif‐ZSM‐30813_ai2mu) data sets. The red line in the schemes indicates the plane from which the single images originate in the CT scan: (a) CT image shows the anterior region of the rostrum with the dorsal group of ampullae of Lorenzini, marked in yellow, transversal cut; (b) the connection between the dorsal and the ventral group of ampullae of Lorenzini via the subnasal fenestrae, transversal cut; (c) the ventral group of ampullae of Lorenzini, marked in pink in the anterior optic region, transversal cut; (d) both lateral groups of ampullae of Lorenzini in the branchial region, marked in green, transversal cut; (e) lateral view of some representatively reconstructed ampullae of Lorenzini of each area and the two innervating branches of the *Nervus trigeminalis* (*Nervus maxillaris* and *Nervus buccalis*) (chondrocranium displayed translucent); (f) dorsal view of the chondrocranium with some representatively reconstructed ampullae of Lorenzini of each area and the *Nervus trigeminalis* (chondrocranium displayed translucent); (g) lateral view of the whole specimen with some representatively reconstructed ampullae of Lorenzini of each area (outer surface displayed translucent); (h) lateral view of the chondrocranium with some representatively reconstructed ampullae of Lorenzini of each area. Abbreviations: II, fossa of the cranial nerve II, VII, *Nervus buccalis*; V_max_, *Nervus maxillaris*; cc, chondrocranium; dal, dorsal ampullae of Lorenzini; kp, keel process; lal, lateral ampullae of Lorenzini; or, olfactory rosette; sf, subnasal fenestrae; val, ventral ampullae of Lorenzini

Generally, the ampullae of Lorenzini open by a dermal porus to the exterior, which receives the electrical stimuli. From these pores, a duct leads into the terminal diverticulum/ampulla (Wueringer, 2012; Wueringer et al., 2021). This in turn consists of several separate circularly arranged chambers in which the signal conversion takes place (Figure 15a–c). A few ampullae and their ducts were segmented and visualized representatively for each region (Figure 15). The majority of ampullae are located along the dorsal area of the rostrum, above the chondrocranium (Figure 15a). They extend from the rostral process until about the middle of the otic region. Their ducts leading to the ampullae are relatively variable in length but tend to be somewhat shorter than those of the other regions (Figure 15a,e–h). The ducts of the ampullae of Lorenzini on the ventral side are generally longer compared to those of the dorsal side but significantly more uniform in overall size (Figure 15c). The ventral and dorsal region of the rostrum initially appear separate from each other in the distal region but are connected centrally behind the olfactory bulbs, between the olfactory trunks (Figure 15b). This connection is reflected in the cranium in the rostral or subnasal fenestra. The ampullae of Lorenzini are embedded in diffuse connective tissue. The dorsal and ventral areas forming the supraorbital or rostral ampullae are innervated by the superficial ophthalmic branches of the cranial nerves V (*N. trigeminus*), which originates behind the vascular sac in the lower part of the metencephalon (Figure 15e,f). Two further areas with ampullae of Lorenzini are located laterally, originating at the level of the suborbital keel process and extend laterally along the surface and the *Musculus adductor mandibulae* muscles to the posterior ends (Figure 15d–h). These so‐called infraorbital ampullae are innervated by the *Nervus maxillaris* and *Nervus buccalis* (Figure 15e,f). The ducts of the ampullae of Lorenzini can become very long in this area, sometimes twice as long as the ducts located at the dorsal and ventral sides of the rostrum (Figure 15e–h).

## 4 DISCUSSION

### 4.1 Comparison of CT examinations to previous anatomical studies

Comparative anatomical studies of Etmopteridae and sharks, in general, are low in number, however, our data allow us to observe characteristic differences of *Etmopterus* described, for example, in Shirai (1992b) to other squaliform sharks in general and within etmopterids in particular.

The anterior fontanelle of the ectethmoid chamber, characteristic of all Etmopteridae, (Shirai & Nakaya, 1990b) can be identified in Figure 3. The absence of character 104 from Shirai (1992b) (Character 104: the presence of *Adductor m. superficialis ß*) allows us to assign our specimen of *E. lucifer* to a group of species, which can be assigned to the *E. lucifer* and *E. pusillus* clades (Straube et al., 2010). The presence of the *Adductor m. superficialis ß* may therefore be synapomorphic for the *E. spinax* and *E. gracilispinis* clades. However, a larger number of samples comprising other species of all four *Etmopterus* clades (Straube et al., 2010) should be considered. The dorsally located suborbital keel process (Figure 3a) appears broad and compact, typical for Etmopteridae (Shirai & Nakaya, 1990a). Another characteristic of *Etmopterus* along with *Euprotomicrus*, *Squaliolus* and *Trigonognathus* identifiable on our CT data is a separate fossa for the external rectus muscle (Shirai & Okamura, 1992).

The branchial arches of *E. lucifer* consist of five pairs of arches, typical for most extant sharks species (Shirai, 1992a). In addition to Shirai's examination of cartilage elements and muscular parts, it was also possible to show and describe essential elements of various sensory organs and the associated nervous system. Especially the brain, the olfactory rosette (containing the olfactory epithelia) and the ampullae of Lorenzini were examined in detail. These structures are often difficult to identify in dissection, as they easily collapse if not supported by surrounding tissue when not prepared in water. Compared to the subadult *E. spinax* analysed in Holmgren (1940), the skull of the *E. lucifer* investigated herein is straighter and more elongated. The skull of the subadult specimen in Holmgren (1940) describes an almost semicircular arc from anterior to posterior (see Holmgren, 1940 fig. 95). Furthermore, the rostrum of *E. lucifer* appears significantly wider compared to the *S. acanthias* specimen studied in Marinelli and Strenger (1959) and Shirai (1992b), whereby the specimen examined by Marinelli and Strenger (1959) was subadult as well and significantly smaller compared to the specimen analysed herein.

The skull cartilage of *E. lucifer* shows a smooth transition from the nasal capsules to the tip of the rostrum and has only a small, separated area towards the rostral process and tapers at this point (Figure 3c,d). The rostral process is more prominent and larger in *S. acanthias*. The nasal capsules of *E. lucifer* occupy a substantial part of the anterior part of the chondrocranium and are significantly larger than those of *S. acanthias*. They are generally more similar to those of *Cirrhigaleus barbifer* (Shirai, 1992b). The rostral or subnasal fenestrae of *E. lucifer* appear significantly larger than those of *S. acanthias* shown in Marinelli and Strenger (1959) and Shirai (1992b). In contrast to the juvenile *E. spinax* (Holmgren, 1940), the rostral fenestrae are not spanned by several cartilage arches but show a smooth opening at the edges (Figure 3c,d). Whether this multiple division is characteristic of *E. spinax* or characteristic for different ontogenetic stages cannot be determined here but requires more specimens (of different sizes) for comparisons. Between nasal capsules and the antorbital shelf, the chondrocranium is incised significantly deeper compared to *S. acanthias* and the antorbital shelf is more extended and thus encapsules the eyeballs wider than in *S. acanthias*. In general, the eye sockets of our specimen  are larger than those of *S. acanthias* but are relatively similar dimensioned in the juveniles of *E. spinax* in Holmgren (1940).

The *foramen nervi II* in *E. lucifer* is more prominent than in *S. acanthias* (Marinelli & Strenger, 1959). The *fossa parietalis* extends posteriorly within the skull cartilage in two relatively short tubular cavities and two lobe‐like branches within the otic region. The depression of the *foramen arteriae carotis internae* is also visible in the model and corresponds in position and size to that of *S. acanthias* (Marinelli & Strenger, 1959; Shirai, 1992b), as well as that of *E. spinax* (Holmgren, 1940). The basicranial keel process is a prominent outgrowth shortly in front of the basal angle. In Etmopteridae it persists until the adult stage, but in our specimen, it appears much wider and more massive than the ones of *Aculeola* and *Centroscyllium* (Shirai, 1992b; Shirai & Nakaya, 1990b). In the *E. lucifer* examined here this keel process serves at the base, in combination with the interorbital wall, as an attachment site for the *Musculus suborbitalis*, which is relatively prominent. According to Shirai (1992b), the muscle fibres inserted into the palatoquadratum in the family Etmopteridae should not be distinguishable from the *M. adductor mandibulae*. In the present case, however, the suborbital muscle is very distinct and clearly separated from the mandibular adductor muscle. This muscle seems to be generally used to protrude the jaws. In this way, it is possible to extend the mouth opening. This may be advantageous for feeding or scavenging on larger prey items to saw out pieces, which is a common feeding strategy taking into account the heterodont dignathy and stomach content analyses of *E. spinax* (Neiva et al., 2006).

### 4.2 Anatomical adaptations to the deep sea

As indicated by previous studies, inter‐specific variation in the morphology, quantity and distribution of ampullae of Lorenzini and their associated structures cannot be strictly correlated with morphological similarities or a close phylogenetic relation, but rather with feeding ecology and habitat (Kajiura et al., 2010; Kempster et al., 2012, 2013). In line with these results, further cranial characteristics may also reflect adaptations to the (deep‐sea) habitat of *Etmopterus*. In fact, the head anatomy suggests that the olfactory system in combination with the ampullae of Lorenzini are the dominant senses likely used for prey detection. Both are notably enlarged in comparison to *S. acanthias*, a squaliform not permanently inhabiting the deep sea (Ebert et al., 2021).

Kajiura et al. (2010) describe the dorsal‐to‐ventral pore distribution ratio for multiple shark species. *Etmopterus lucifer* and *S. acanthias* show a rather similar distribution. Both species exhibit more pores on the ventral side (approx. ratio dorsal–ventral pore distributions: 0.6 vs. 0.5). This may suggest that both species feed rather benthos orientated or generally tend to approach their prey from above. Considering the ratios of species that can be confidently classified as benthic feeders (e.g. *Ginglymostoma cirratum*, *Stegostoma fasciatum*, *Carcharias taurus*), the ratios are remarkably similar. Therefore, it can be assumed that *E. lucifer* has a similar pore distribution because of its feeding habits. Also, Kajiura et al. (2010) state that deep sea‐inhabiting species with large pore numbers (*E. lucifer* possesses one of the highest pore numbers of all species in the study) are likely forageing off the sea floor. *Etmopterus lucifer* has almost four times more ampullae of Lorenzini compared to other species studied by Kajiura et al. (2010). The high number of electroreceptors could facilitate the detection of fast and agile prey, especially in the open water column (Kajiura et al., 2010) as prey items of this opportunistic feeder also encompasses squids and myctophid fishes (Martin & Mallefet, 2022). Further, pores and ducts associated with transporting electric signals to synapses with a larger diameter, which are often found in deep‐sea sharks, reduce the electrical impedance along the length of the duct. This probably results in increased sensitivity. This may be an advantage of comparatively small‐bodied deep‐sea shark species, as a similar electrosensory sensitivity is achieved as in larger‐bodied species (Kajiura et al., 2010).

For highly migratory species, specific senses for orientation and navigation are essential, especially in the pelagic realm lacking location‐specific landmarks (Meredith et al., 2022). The use of the geomagnetic field as a navigation aid in chondrichthyans was presumed previously (Kalmijn, 1974; Klimley, 1993; Paulin, 1995). Experiments in *Urobatis jamaicensis* and juvenile *Sphyrna tiburo* strengthened the assumption that there is an active use of the geomagnetic field of the earth in these species (Keller et al., 2021; Newton & Kajiura, 2020). It is not yet fully clarified whether the detection of variation in magnetic fields is conducted via the ampullae of Lorenzini or/and through another (unknown) structure (Anderson et al., 2017; Kalmijn, 1984; Walker et al., 2003). *E. lucifer* has very long ampullar channels, especially on the lateral sides of the head. According to Sisneros and Tricas (2002) the sensitivity of the receptor is directly correlated to the length of the ampullar duct. Since the geomagnetic field is relatively weak, high sensitivity would be needed to detect it. Therefore, those ampullae of Lorenzini with distinctly long ducts may qualify for functioning as a navigation aid using the geomagnetic field. However, no migratory behaviour is documented in *Etmopterus* so far. Nevertheless, several molecular studies suggest large distribution ranges for some *Etmopterus* species (Agne et al., 2022; Straube, Duhamel, Gasco, Kriwet, & Schliewen, 2011; Straube, Kriwet, & Schliewen, 2011; Straube et al., 2021) which may be explained by migratory behaviour.

*Etmopterus lucifer* (158–1357) occurs in a greater median depth than *S. acanthias* (0–600 m) (Ebert et al., 2021). At these depths, short wavelengths predominate, i.e. blue light and only a small number of photons is still present (Lisney et al., 2012). Enlarged eyes frequently evolved in various species inhabiting such depths. In addition to the enlargement for increased photon sensitivity, sensitivity is also shifted to specific wavelengths (Meredith et al., 2022). That increased sensitivity helps on one hand to efficiently use the low amount of penetrating light and on the other hand to detect blue light emitted via the bioluminescence of prey and the light emission of conspecifics' photophores (Claes et al., 2014; Ebert et al., 2021; Reif, 1985). Kajiura et al. (2010) show that the eye diameter of sharks increases with the depth of occurrence. Deep‐sea species seem to have larger eye diameters than shark species found in shallower habitats. In fact, *E. lucifer* shows the largest relative eye diameter of all sharks studied in Kajiura et al. (2010). This all together may indicate that the sense of sight for *E. lucifer* is higher developed than in other species of the Etmopteridae investigated in Kajiura et al. (2010) and certainly more emphasized than in *S. acanthias*.

The dimensions of the olfactory bulbs were often used to determine the olfactory capacity of a species and would indicate the significance of the olfactory sense in *E. lucifer* (Camilieri‐Asch, Shaw, et al., 2020; Camilieri‐Asch, Yopak, et al., 2020; Schluessel et al., 2008, 2009; Theisen et al., 1986; Zeiske et al., 1987). The sheer size of the nasal capsules does not necessarily allow conclusions on the efficiency of the olfactory system (Gardiner et al., 2012; Meredith et al., 2012; Meredith & Kajiura, 2010), however, Camilieri‐Asch, Yopak, et al. (2020) claim that the dimensions of the olfactory bulbs are correlated with the number of olfactory stimuli through the epithelium. They conclude that the dimensions can be considered a valid proxy for olfactory ability in elasmobranchs. In contrast, other studies state that the size of the olfactory bulbs likely reflects rather a functional specialization taking the habitat and ecology of species into account (Kotrschal et al., 2017; Schluessel et al., 2008; Theiss et al., 2009; Yopak et al., 2015). The functional adaptation matches with results from Yopak et al. (2015), where the standardized residual olfactory bulb size of *E. lucifer* is consistent with the general trend that deep‐sea shark species have large olfactory bulbs, showing a presumably greater reliance on the olfactory sense in environments with low visual cues. The brain areas responsible for processing signals from the olfactory sense and the receptor cells of the ampullae of Lorenzini are notably enlarged compared, for example, to *S. acanthias* (Figure 11) further supporting the increased functionality of these senses in a species permanently inhabiting deep‐sea habitats.

Several studies showed that galeomorph sharks and myliobatiform rays tend to have larger brains than squalomorph sharks and holocephali (Myagkov, 1991; Northcutt, 1978; Yopak & Lisney, 2012). In general, especially deep‐sea inhabiting species show average to below‐average brain dimensions in relation to their body size compared to species that tend to live in shallower habitats (Yopak & Montgomery, 2008). According to Kajiura et al. (2010), the relative brain size of *E. lucifer* fits with that trend across other shark species. Characteristic of deep‐sea Chondrichthyes is an enlarged medulla oblongata and cerebellar‐like structures (i.e. the auricles of the cerebellum, formed by two areas of granule cells) and a reduced telencephalon. The cerebellar‐like structures are processing electroreception input from the ampullae of Lorenzini (transmission of stimulus via the anterior lateral line nerve) (Yopak, 2022). The enlargement of the cerebellar‐like structures in *E. lucifer* additionally underlines the importance of the electroreception and the lateral line system of this species which is formed by the lateral granular area and the dorsal granular ridge. This is in contrast to the shallower occurring *S. acanthias*, which does not show any enlargement of the cerebellar‐like structures (Kajiura et al., 2010).

In summary, this suggests that olfactory and optical senses as well as the detection of electrical stimuli by the ampullae of Lorenzini show distinct functional adaptations in lantern sharks and are likely ecomorphological adaptations to their habitat.

## 5 CONCLUSIONS

This study describes the cranial morphology of an etmopterid shark species in detail using (dice)micro‐CT data for the first time. Camilieri‐Asch, Caddy, et al. (2020) and Camilieri‐Asch, Shaw, et al. (2020) have also performed diceCT scans, but of galeomorph sharks, among others, and published the results in two very recent studies. Kamminga et al. (2017) performed CT scans of a wide variety of species but included in their database only hard tissues. In contrast, we provided a detailed investigation of this *Etmopterus lucifer* comprising skeletal elements but also soft tissues such as muscles, nerves and multiple sensory systems. Our data are based on iodide staining of a 39‐year‐old formaldehyde fixed and EtOH‐preserved wet‐collection specimen of *Etmopterus lucifer*. The data allow us to identify important hard tissues as well as muscles and sensory structures and their connections to the brain in detail and allow comparison with results of previous studies. The most striking sensory structures are the ampullae of Lorenzini, the olfactory and visual sensory systems, which appear to represent the dominant sensory organs in this species. Their morphology reflects functional adaptations to the deep‐sea habitat of *E. lucifer* which is further supported by the morphology of the corresponding brain areas. The content of the micro‐CT reconstructions of *E. lucifer*, will serve as a basis for future studies and may be the first step for a series of comparative works on the cranial morphology of squaliform sharks.

## AUTHOR CONTRIBUTIONS

M.A.S., B.R. and N.S. conceived the study. B.R. and M.A.S. performed the specimen pre‐treatment (iodine staining) and B.R. the CT scanning. M.A.S. and B.R. processed the scan data. M.A.S. performed the 3D analysis and wrote the anatomical description. B.R. and N.S. edited and added to the manuscript. All authors approved the final version.

## CONFLICT OF INTEREST

The authors declare that they have no conflict of interest.

## Data Availability

All figures are included and visible in the submission.
